# Doomscrolling, mental health, and psychological well-being in adolescents and adults: a systematic review and meta-analysis

**DOI:** 10.3389/fpsyg.2026.1893800

**Published:** 2026-09-18

**Authors:** Qi Li, Xinrui Yin

**Affiliations:** Department of Anesthesiology, Aerospace Center Hospital, Beijing, China

**Keywords:** doomscrolling, doomsurfing, mental health, meta-analysis, psychological distress, psychological well-being, systematic review

## Abstract

**Background:**

Doomscrolling is persistent or repetitive engagement with predominantly negative, threatening, or distressing online information, often accompanied by difficulty disengaging. It overlaps with, but is not interchangeable with, problematic social media use, general negative-news exposure, or broader problematic internet use. We synthesized associations between doomscrolling and mental-health or psychological well-being outcomes.

**Methods:**

This PROSPERO-registered systematic review and meta-analysis (CRD420261395295) followed PRISMA 2020. Searches covered nine bibliographic, registry, grey-literature, and citation sources through 20 May 2026. Article- or supplement-reported zero-order Pearson or Spearman correlations were synthesized using Fisher’s z transformation and random-effects models with restricted maximum likelihood estimation and Hartung–Knapp adjustment. Adjusted regression or path coefficients and duplicated same-sample scale versions were not pooled. Design-appropriate JBI checklists and GRADE were used for critical appraisal and certainty assessment.

**Results:**

Forty-four reports representing 44 unique studies were included. Twenty-one studies (23 independent analytic samples) contributed 40 effects to the five primary meta-analyses; the remaining 23 reports contributed narrative or nonprimary quantitative evidence. Doomscrolling was associated with anxiety (*r* = 0.35, 95% CI 0.27–0.43), depression (*r* = 0.35, 95% CI 0.16–0.52), psychological distress (*r* = 0.51, 95% CI 0.37–0.62), lower psychological well-being (*r* = −0.29, 95% CI − 0.38 to −0.20), and lower life satisfaction (*r* = −0.25, 95% CI − 0.37 to −0.13). Heterogeneity was high in every domain; for psychological distress, *I*^2^ was 86.5% and four of five contributing studies were from Türkiye. Certainty was very low for all five outcomes.

**Conclusion:**

Doomscrolling is associated with poorer mental-health and well-being outcomes, but the evidence does not establish causality. Cross-sectional designs, high heterogeneity, residual confounding, and geographic concentration limit inference. Standardized measurement, longitudinal and ecological momentary assessment studies, experiments, and preregistered cross-cultural research are needed.

**Systematic review registration:**

https://www.crd.york.ac.uk/PROSPERO/view/CRD420261395295, identifier PROSPERO (CRD420261395295).

## 1 Introduction

The contemporary digital information environment exposes users to continuous streams of negative, threatening, and emotionally charged content. Doomscrolling refers to persistent or repetitive engagement with predominantly negative, threatening, or distressing online information, commonly through scrolling, together with difficulty disengaging despite an unpleasant emotional experience (Sharma et al., 2022; Satici et al., 2023). This content-and-engagement pattern distinguishes doomscrolling from problematic social media use, which centers on dysregulated platform use and need not involve negative content; from general negative-news exposure, which captures content exposure without necessarily implying repetitive scrolling or impaired disengagement; and from problematic internet use, which is broader and content-nonspecific. These constructs and adjacent behaviors can overlap, so the distinction is conceptual rather than an assertion of complete independence.

Empirical work has rapidly expanded beyond the original Doomscrolling Scale. Full-length, short-form, translated, and platform-specific measures now operationalize related forms of repetitive negative-content engagement (Sharma et al., 2022; Satici et al., 2023; Shabahang et al., 2024; Shabahang et al., 2023). Recent studies illustrate both convergence and boundary questions. Soraci et al. validated both the DSS-15 and DSS-4 in an Italian sample; the reported psychological associations used in the present outcome syntheses were DSS-15 associations, whereas no DSS-4 criterion-correlation matrix suitable for the primary outcome pools was reported (Soraci et al., 2025). Türk-Kurtça and Kocatürk examined trait anxiety, intolerance of uncertainty, psychological resilience, and doomscrolling (Türk-Kurtça and Kocatürk, 2025). Dominguez-Rodriguez et al. distinguished general doomscrolling from climate-change-specific doomscrolling, illustrating how a domain-specific negative-news exposure can overlap with, but should not automatically be treated as identical to, the general construct (Dominguez-Rodriguez et al., 2025).

Three complementary process families motivate the outcome domains examined here. First, threat and uncertainty processes may connect repetitive negative-information monitoring with anxiety. Second, negative affect, rumination, and other forms of perseverative engagement may correspond to depression and broad psychological distress. Third, repeated allocation of attention and self-regulatory resources to distressing content may be associated with lower psychological well-being and life satisfaction. These pathways are theoretically plausible and consistent with correlational patterns, but the available evidence does not establish them as causal mechanisms.

Existing narrative and scoping reviews have clarified definitions, proposed mechanisms, and mapped the early empirical literature on doomscrolling and mental health (Güme, 2024; Sharpe et al., 2026). A recent PRISMA-ScR review synthesized 17 empirical studies narratively. Broader syntheses of digital technology, screen use, social media, or technology-related stress provide useful context (Lițan, 2025), but they combine heterogeneous exposures and do not isolate repetitive engagement with negative online information. Outcome-specific pooled estimates and an explicit assessment of certainty therefore remained needed.

We synthesized empirical evidence on associations between doomscrolling and anxiety, depression, psychological distress, psychological well-being, and life satisfaction in adolescent and adult populations. The review distinguished poolable article- or supplement-reported zero-order correlations from adjusted, domain-specific, duplicated, or otherwise nonprimary evidence; quantified heterogeneity and prediction intervals; evaluated risk of bias and certainty; and used sensitivity analyses to examine trait versus symptom anxiety, suspected sample overlap, low-risk-of-bias evidence, measurement type, and geographic concentration.

## 2 Methods

### 2.1 Protocol and registration

This systematic review and meta-analysis was conducted in accordance with the Preferred Reporting Items for Systematic Reviews and Meta-Analyses 2020 statement (Page et al., 2021) and reported the search strategy following the PRISMA-S 2021 extension (Rethlefsen et al., 2021). The protocol was prospectively registered in the International Prospective Register of Systematic Reviews under the registration number CRD420261395295.

This review synthesizes empirical evidence on the association between doomscrolling and mental health and psychological well-being outcomes in adolescents and adults. The prespecified focus was on doomscrolling as a distinct maladaptive pattern of digital media use characterized by persistent engagement with negative or distressing online content, rather than general social media use, problematic social media use, smartphone use or smartphone addiction, news consumption, short-form video addiction, or cyberchondria. The eligibility criteria, outcome-level synthesis strategy, handling of non-poolable studies, and planned sensitivity analyses were specified in the registered protocol before quantitative synthesis.

### 2.2 Eligibility criteria

Eligibility criteria were defined according to a PECO framework. The population of interest comprised adolescents and adults, including university students, young adults, general community samples, employees, healthcare workers, teachers, and social media users. Studies were eligible when all participants were aged 16 years or older, or when the sample mean age was 16 years or older with no more than 20% of participants below age 16. Studies focusing exclusively on children or samples with a mean age below 16 years were excluded.

The exposure of interest was doomscrolling or doomsurfing, including orthographic variants. Eligible measures assessed persistent or repetitive engagement with predominantly negative, threatening, or distressing online content as a distinct construct. General social media use, problematic social media use, smartphone use, broad problematic internet use, general news consumption, cyberchondria, and short-form video overuse were not treated as substitute exposures. Domain-specific doomscrolling measures could inform narrative or sensitivity analyses but were not co-entered with a general doomscrolling measure from the same sample in the same primary pool.

Primary outcome domains were depression, anxiety, psychological well-being, life satisfaction, and psychological distress. Secondary outcomes included perceived stress, sleep quality or insomnia, loneliness, burnout, secondary traumatic stress, post-traumatic stress symptoms, positive and negative affect, self-esteem, emotion regulation, and fear of missing out. Outcome domains with at least three eligible studies were considered for quantitative synthesis, whereas outcome domains represented by fewer than three eligible studies were synthesized narratively.

Eligible designs included cross-sectional, longitudinal, cohort, daily diary or experience-sampling, experimental, and psychometric validation studies reporting an association between doomscrolling and at least one eligible psychological outcome. Primary meta-analysis required an article- or supplement-reported zero-order Pearson correlation, Spearman correlation, or sufficient aggregate information to derive an interpretable correlation. Adjusted regression coefficients, path coefficients, experimental contrasts, and zero-order correlations recomputed solely from deposited individual-level data were not promoted to the primary pools. Conceptually eligible reports without a qualifying primary effect were retained for narrative or nonprimary quantitative synthesis.

### 2.3 Information sources and search strategy

The systematic literature search was conducted in PubMed, Embase, Web of Science, and Scopus on 20 May 2026, with no restriction on publication year. Searches were also conducted in the Cochrane Library (including CENTRAL), CNKI, and ClinicalTrials.gov to identify clinical trial records, Chinese-language studies, and registered or unpublished protocols. Google Scholar was searched as a supplementary source for grey literature and recently posted records; the first 200 results sorted by relevance were screened for eligibility and deduplicated against the main database records.

The search strategy used the six orthographic variants of doomscrolling and doomsurfing identified in the eligibility criteria, applied as free-text terms in title, abstract, and, where supported, keyword fields. For PubMed, Web of Science, and Scopus, the doomscrolling exposure block was combined with a broad outcome block covering mental health, depression, anxiety, stress, distress, well-being, life satisfaction, loneliness, sleep, trauma, secondary traumatic stress, post-traumatic stress, and burnout. For Embase, the Cochrane Library, CNKI, and ClinicalTrials.gov, the doomscrolling exposure block was used alone without an outcome filter. This database-specific approach was intended to maximize sensitivity in databases with smaller indexed volume given the construct’s recent emergence and inconsistent indexing, while maintaining precision in the largest databases. Chinese-language equivalents covering five orthographic variants of doomscrolling and the compulsive browsing of negative online news were applied in CNKI.

No publication-status filter was applied at the retrieval stage. No language restriction was applied. Database-specific search strings, field codes, retrieval counts, and supplementary search procedures are reported in Supplementary Methods 1.

### 2.4 Study selection

All records retrieved from the database searches were exported and merged for deduplication. Duplicate records were identified using a combination of automated matching by DOI, PMID, or other unique identifiers when available, and manual verification by title, author, and publication-year matching. Records from Google Scholar were managed as a supplementary source and were deduplicated against the main database records before full-text assessment. Backward and forward citation chasing was performed by hand-searching the reference lists of all studies meeting full-text eligibility and by screening forward citations of the original Doomscrolling Scale paper; no additional eligible studies were identified through this process.

After deduplication, titles and abstracts were independently screened by two reviewers against the predefined eligibility criteria. Records that clearly did not address doomscrolling or doomsurfing, did not include any of the prespecified primary or secondary outcomes, or were reviews, commentaries, protocols, or non-empirical publications were excluded. Records that appeared eligible or could not be confidently excluded on the basis of title and abstract were retrieved for full-text assessment. Discrepancies were resolved by discussion until consensus.

Full texts were then independently assessed by two reviewers to determine eligibility for quantitative synthesis or narrative synthesis. Studies were classified as eligible for meta-analysis when they reported an extractable zero-order correlation between doomscrolling and an eligible outcome. Studies that met the conceptual eligibility criteria but lacked extractable correlation coefficients were retained for narrative synthesis. Reasons for exclusion at the full-text stage were documented, and the overall study selection process was recorded according to PRISMA 2020.

### 2.5 Data extraction

Data were extracted independently by two reviewers using a predefined extraction form. Discrepancies were resolved by discussion until consensus. Extracted study-level information included first author, publication year, country or region, study design, recruitment setting, sample size, population type, age, sex distribution, publication status, and peer-review status. Exposure-related information included the doomscrolling measure used, scale version, number of items, response format, scoring direction, and internal consistency reliability (Cronbach’s α or McDonald’s ω) when reported.

Outcome-level data were extracted in long format at the study–outcome level. For each eligible outcome, we recorded the outcome domain, measurement instrument, effect-size type, correlation coefficient, sample size used for the effect estimate, *p* value or confidence interval when reported, and whether the estimate was a zero-order or adjusted association. Methodological information relevant to risk-of-bias assessment, including sampling method, confounding adjustment, measurement validity, and information for identifying overlapping samples, was also extracted from each report.

When multiple eligible outcomes were reported, associations were extracted at the study–sample–outcome level. Within the same sample and outcome domain, duplicated scale versions were not co-entered: the full or general doomscrolling measure was prioritized over a short or platform-specific version, and a domain-specific exposure was not entered alongside the general measure in the same pool. Article- or supplement-reported zero-order Pearson or Spearman correlations were prioritized; adjusted regression and path coefficients were retained narratively. Authors were not contacted for missing data. If outcome scoring direction or polarity could not be verified from the report or supplement, the effect was not reverse-coded by assumption and was retained narratively.

### 2.6 Risk of bias and certainty of evidence assessment

All 46 appraisal units, including evidence retained narratively or nonprimarily, were assessed independently by two reviewers using the design-appropriate Joanna Briggs Institute checklist (Aromataris and Munn, 2020). Analytical cross-sectional studies used the 8-item JBI Analytical Cross-Sectional Checklist (2020 version), cohort or longitudinal designs used the 11-item JBI Cohort Checklist, and randomized experiments used the 13-item JBI RCT Checklist. Disagreements were resolved by discussion. Item-level judgments were Yes, No, or Unclear; no Not-applicable judgments occurred.

JBI does not prescribe an overall Low/Moderate/High score, so item-level judgments remained primary. For sensitivity and summary purposes, we used a review-level derived classification harmonized across checklist lengths: at least 87.5% affirmative applicable items was classified as low risk, 62.5% to less than 87.5% as moderate risk, and less than 62.5% as high risk. Thus, the equivalent thresholds were 7–8/8, 10–11/11, and 12–13/13 for low risk; 5–6/8, 7–9/11, and 9–11/13 for moderate risk; and 0–4/8, 0–6/11, and 0–8/13 for high risk. These were review-specific summaries rather than official JBI scores, and no discretionary overrides were applied. Full item-level judgments appear in Supplementary Table S2.

Certainty for each primary outcome was assessed with GRADE (Guyatt et al., 2011). Because the evidence was observational and predominantly cross-sectional, certainty started at low and was considered for downgrading for risk of bias, inconsistency, indirectness, imprecision, and publication bias. Upgrading required a large, consistent association, dose–response evidence, or a credible pattern in which residual confounding would attenuate the association. Certainty judgments and reasons are reported in Supplementary Table S3.

### 2.7 Effect size calculation

The primary effect size was the zero-order correlation coefficient between doomscrolling and each eligible psychological outcome. Pearson’s r was extracted when available. Spearman’s rho was also eligible when reported as the only correlation estimate and was treated as an approximation of the corresponding correlation for synthesis; a sensitivity analysis excluding Spearman-based estimates was conducted. Correlations were extracted in their reported direction: positive coefficients indicated that higher doomscrolling was associated with higher scores on the outcome measure, whereas negative coefficients indicated an inverse association. Thus, for adverse outcomes such as depression, anxiety, stress, and psychological distress, positive correlations indicated greater symptom burden, whereas for adaptive outcomes such as psychological well-being and life satisfaction, negative correlations indicated lower well-being. When the scoring polarity or direction of an outcome could not be verified from the article or Supplementary material, no assumption-based sign reversal or parameter recoding was undertaken; the effect was retained for narrative synthesis.

For quantitative synthesis, correlation coefficients were transformed to Fisher’s z values before pooling. The sampling variance was calculated as 1/(*n* − 3), where n represented the sample size contributing to the correlation estimate. Pooled Fisher’s z estimates were back-transformed to correlation coefficients for interpretation. When confidence intervals or standard errors were not directly reported, they were derived from the Fisher’s z variance where sufficient sample-size information was available.

Standardized or unstandardized regression coefficients, mediation or structural-path coefficients, multilevel estimates, and experimental effect sizes were not treated as interchangeable with zero-order correlations. Public-data-derived correlations not printed in the report or supplement were not included in primary synthesis. These rules prevented adjusted effects and duplicated general/domain-specific exposures from entering the same primary pool.

### 2.8 Data synthesis and statistical analysis

Quantitative synthesis was performed separately for anxiety, depression, psychological distress, psychological well-being, and life satisfaction. Across these domains, 21 studies contributed 23 independent analytic samples and 40 primary effects. Outcome domains without sufficient comparable zero-order correlations were summarized narratively or as nonprimary quantitative evidence.

For each outcome domain, Fisher’s z-transformed correlations were pooled using a random-effects model. A random-effects approach was selected because between-study heterogeneity was expected due to differences in populations, cultural settings, doomscrolling measures, and outcome instruments. Between-study variance was estimated using restricted maximum likelihood. Confidence intervals for pooled effects were calculated using the Hartung–Knapp adjustment to provide more robust uncertainty estimates, particularly for outcome domains with a small number of studies (Hartung and Knapp, 2001). Pooled estimates were back-transformed from Fisher’s z to the r scale for presentation and interpretation.

Statistical heterogeneity was assessed using τ^2^, *I*^2^, and Cochran’s Q. Where at least three studies were available, 95% prediction intervals were also calculated to indicate the expected range of effects in a future comparable study (IntHout et al., 2016). A summary forest plot was generated to display pooled estimates, 95% confidence intervals, 95% prediction intervals, and heterogeneity statistics across the primary outcome domains. When more than one eligible effect size from the same study contributed to the same outcome domain, dependency was handled according to the prespecified rules described below.

All analyses were performed in R using the metafor package (Viechtbauer, 2010), with cluster-robust variance estimation implemented through the clubSandwich package (Pustejovsky and Tipton, 2018). Results were interpreted primarily in terms of effect magnitude, direction, confidence intervals, prediction intervals, heterogeneity, and certainty of evidence.

### 2.9 Dependent effect sizes and sample overlap

Potential dependency was considered within and between reports. Within a sample and outcome domain, the most general, full-length, and best-validated doomscrolling measure was prioritized, with alternate versions reserved for sensitivity or narrative assessment. Multiple independent samples within a report were retained as separate analytic samples when recruitment and reporting supported independence.

Between-report dependency was assessed using author overlap, country and recruitment setting, sample size, demographic characteristics, data-collection period, ethics information, survey platform, and study context. No confirmed duplicate primary sample was identified. One suspected overlap involved the two 2025 reports associated with the Kurtça/Türk-Kurtça author team; both were retained in the main anxiety analysis and examined through alternate-exclusion sensitivity analyses. Details of the overlap assessment and final adjudication are provided in Supplementary Table S4.

The main analyses therefore retained all eligible independent primary effects. Additional exploratory sensitivity analyses excluded the trait-anxiety effect, alternately removed each report in the suspected-overlap pair, restricted analyses to low-risk-of-bias units, and examined the geographic concentration of psychological distress.

### 2.10 Sensitivity, subgroup, and publication bias analyses

Robustness checks included leave-one-out analysis, exclusion of Spearman-based estimates, restriction to low-risk-of-bias appraisal units, exclusion of the Türk-Kurtça trait-anxiety effect, alternate exclusions within the suspected-overlap pair, and a geographic concentration analysis for psychological distress. These additional analyses were treated as exploratory sensitivity analyses rather than as prespecified primary analyses.

For domains with at least ten primary effects, we also performed an exploratory measure-type comparison between the full-length DSS-15 and the combined DSS-4/SMDS short-form or platform-specific group. This comparison was applied to anxiety and psychological well-being. Directions, group-specific pooled estimates, heterogeneity, and tests of between-group difference were reported; the analysis was not used to assert that measure type explained heterogeneity.

Publication bias and small-study effects were assessed only for outcome domains with at least ten effect sizes. For eligible domains, funnel plots were visually inspected, and Egger’s regression test was used to evaluate asymmetry (Egger et al., 1997). Trim-and-fill analyses were used to examine the potential influence of missing small studies on pooled estimates (Duval and Tweedie, 2000). For outcome domains with fewer than ten effect sizes, publication bias analyses were not conducted due to insufficient statistical power.

### 2.11 Narrative synthesis of non-poolable studies

Conceptually eligible reports or outcomes without a qualifying article- or supplement-reported zero-order correlation were retained for narrative or nonprimary quantitative synthesis. This included adjusted regression and path coefficients, experimental contrasts, correlations derived solely from deposited individual-level data, adjacent constructs, sparse domains, and effects with unverified scoring direction. In particular, the Yang sleep/insomnia association was not reverse-coded by assumption because its polarity could not be reconstructed confidently from the report or supplement.

Narrative synthesis was organized by outcome domain and study design. For each non-poolable study, we summarized the population, doomscrolling measure, outcome construct, direction of association, and whether the finding was broadly consistent with the corresponding pooled outcome domain when such a meta-analysis was available (Campbell et al., 2020). Narrative conclusions were based on the pattern, direction, and methodological context of findings rather than vote counting by statistical significance. These studies were used to contextualize the quantitative findings and to identify evidence gaps, but they were not included in pooled effect estimates unless sufficient zero-order correlation data were available.

## 3 Results

### 3.1 Study selection

Database and register searches identified 190 records. After removal of 82 duplicates and 25 conference abstracts, 83 records were screened; 49 reports were sought, 9 were not retrieved, and 40 reports were assessed for eligibility. Two reports were excluded at full text. The supplementary Google Scholar search contributed six additional eligible reports, while citation chasing contributed none.

Forty-four reports representing 44 unique underlying studies were included (Figure 1). Twenty-one studies, comprising 23 independent analytic samples, contributed 40 effects to the five primary meta-analyses. The other 23 reports contributed narrative or nonprimary quantitative evidence; they were not all narrative-only because some contained quantitative estimates that did not meet the primary pooling rules.

**Figure 1:**
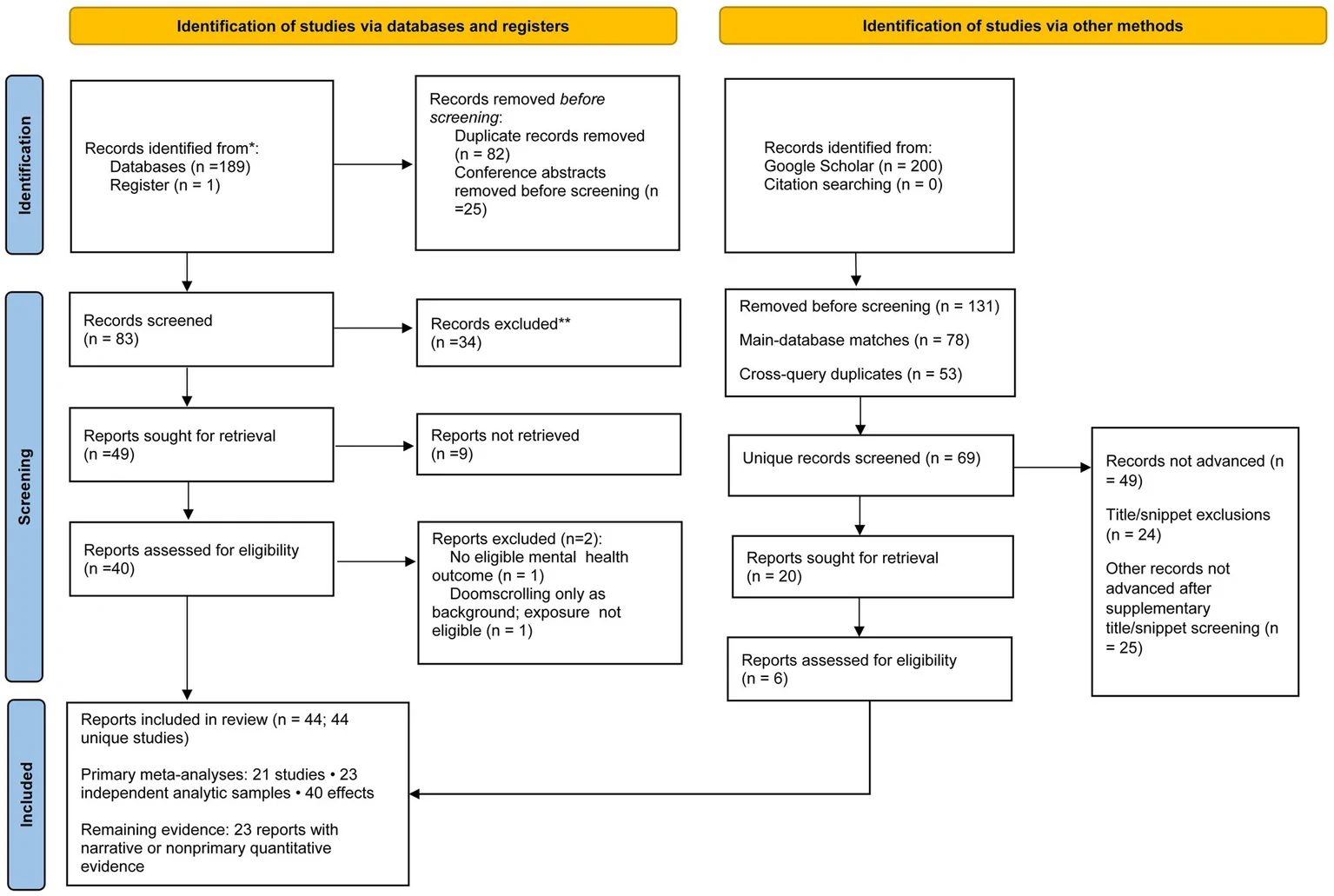
PRISMA 2020 flow diagram of report identification and selection. Forty-four reports representing 44 unique underlying studies were included. Twenty-one studies (23 independent analytic samples) contributed 40 effects to the five primary meta-analyses; the remaining 23 reports contributed narrative or nonprimary quantitative evidence.

### 3.2 Study characteristics

The 44 included reports were published between 2021 and 2026. Structured quantitative fields were available for 30 reports: 21 primary-meta studies and 9 additional reports with quantitative but nonprimary evidence. Fourteen further reports were retained narratively, giving 23 reports outside the primary meta-analyses in total. Table 1 describes the 30-report structured subset without implying that all 30 contributed to primary pooling; full study- and outcome-level information is provided in Supplementary Table S1.

**Table 1:** Characteristics of the 30-report structured quantitative-extraction subset (21 primary-meta studies and 9 reports with nonprimary quantitative evidence).

Report/study (Author, year)	Country/region	Study design	Population	Sample size	Mean age	Female, %	Doomscrolling measure	Main eligible outcome domains	Role in synthesis
Ulaş and İncekara (2026)	Türkiye/Middle East	Cross-sectional	University students	414	NR	47.8%	DSS-15	PTSD, PSMU, OTHER	Quantitative/nonprimary evidence
Camadan and Uzunoğlu (2026)	Türkiye/Middle East	Cross-sectional	Adults	663	25.00	80.1%	DSS-4/short form (DS-SF)	SLEEP, FoMO, OTHER	Quantitative dataset; not pooled
Ang (2025)	Singapore/Southeast Asia	Cross-sectional	Social media users	235	29.81	76.2%	SMDS	ANX, DEP, STR, STS	Meta-analysis
Yıldırım and Toygar (2026)	Türkiye/Middle East	Cross-sectional	Other (maritime deck cadets)	380	NR	8.9%	DSS-4/short form	LS, PA	Meta-analysis
Dominguez-Rodriguez et al. (2025)	Netherlands/International online sample/Multi-region	Cross-sectional	Mixed adult sample	365	25.12	55%	General DSS-15; climate-specific CCDS	ANX, DEP, OTHER	Narrative/nonprimary; adjusted regressions
Yang et al. (2024)	China/East Asia	Two-study cross-sectional	University students	Study 12,885; Study 2,578	19.36 S1; 19.41 S2	39.41% S1; 37.54% S2	Chinese DSS-15/DSS-4	ANX, DEP, SLEEP, OTHER	Partial: DEP/ANX meta; SLEEP narrative
McCutcheon et al. (2024)	Iran; United States/Multi-region	Cross-sectional	University students	812	Iran 25.31; US 20.04	Iran 79%; US 68%	SMDS	ANX, DEP, PWB, OTHER	Meta-analysis
Taskin et al. (2024)	Türkiye/Middle East	Cross-sectional	Social media users	400	NR	NR	DSS-15	PWB, STS, OTHER	Meta-analysis
Kartol et al. (2025)	Türkiye/Middle East	Psychometric validation	Adults	508 (Phase 2 with doomscrolling and outcomes)	NR	NR	DSS-15	PWB, OTHER	Quantitative dataset; not pooled
Kartol et al. (2023)	Türkiye/Middle East	Cross-sectional	Adults	402	NR (young adult majority)	>70%	DSS-15	ANX, PD	Meta-analysis
Satıcı (2023)	Türkiye/Middle East	Cross-sectional	Social media users	Study II 419; Study III 460	SII 24.47; SIII 23.68	Study II 74%; Study III 74.6%	DSS-15 and DSS-4 evaluated; DSS-15 used for the primary associations	PD, PWB, LS, FoMO, SMA, OTHER	Meta-analysis
Buchanan et al. (2021)	United Kingdom/Europe	Experimental	Mixed adult sample	Study 1,299; Study 2,603	S1 29; S2 26	S1 74%; S2 59%	Author-developed experimental exposure	PA, NA, OTHER	Quantitative dataset; not pooled
Antonysamy and Parameswari (2026)	India/South Asia	Cross-sectional	University students	399	20.5 (range)	NR	DSS-15	PWB, SMA, OTHER	Meta-analysis
Kaya and Griffiths (2025)	Türkiye/Middle East	Cross-sectional	University students	496	20.26	74.2%	DSS-4/short form (DS-SF)	PWB, OTHER	Meta-analysis
Soraci et al. (2025)	Italy/Europe	Psychometric validation	Adults	300	NR	NR	DSS-15 primary associations; DSS-4 psychometrically evaluated, but no criterion-correlation matrix suitable for the primary outcome pools was reported.	ANX, DEP, PD, PWB, LS; PSMU/STR nonprimary	Primary meta-analysis
Blacha et al. (2025)	Canada/North America	Cross-sectional	University students	677	19.57	72.2%	DSS-15	LS, OTHER	Meta-analysis
Kaya and Griffiths (2025)	Türkiye/international online/Middle East	Cross-sectional	Adults	432	33.31	70.6%	DSS-4/short form	PWB, OTHER	Meta-analysis
Türk-Kurtça and Kocatürk (2025)	Türkiye/Middle East	Cross-sectional	Adults	553	NR	NR	DSS-15	ANX, SMA, OTHER	Meta-analysis
Shabahang et al. (2023)	Iran/Middle East	Cross-sectional	Social media users	707 (40 outliers removed)	28.38	82.2%	SMDS	ANX, DEP, PWB, LS	Meta-analysis
Körün and Satıcı (2026)	Türkiye/Middle East	Cross-sectional	Adults	811	23.01	NR	DSS-15	PWB, OTHER	Quantitative dataset; not pooled
Shabahang et al. (2024)	Iran; United States/Multi-region	Cross-sectional	University students	Iran 620; US 180	Iran 26.75; US 20.06	Iran 26.6%; US 75.0%	SMDS	ANX, OTHER	Meta-analysis
Kütük et al. (2025)	Türkiye/Middle East	Cross-sectional	University students	406	22.31	82.3%	Brief DSS (TDS-BF)	PWB, OTHER	Meta-analysis
Sharma et al. (2022)	United States (OECD online panel)/Multi-region	Psychometric validation	Social media users	Study 2,401; Study 3,502	NR	NR	DSS-15	ANX, PWB, PA, FoMO, PSMU	Meta-analysis
Hughes et al. (2024)	United Kingdom/Europe	Two-study cross-sectional	Employees	Study 1: 352; Study 2: 126 participants/630 observations	NR	NR	Author-developed single-item Work Doomscrolling measure	OTHER	Quantitative dataset; not pooled
Türk-Kurtça and Kocatürk (2025)	Türkiye/Middle East	Cross-sectional	Adults	443	23.49	79.9%	DSS-15	Trait ANX; IU and resilience nonprimary	Primary anxiety; trait/overlap sensitivities
Biricik et al. (2026)	Türkiye/Middle East	Cross-sectional	University students	779	NR (17–55 + age bands)	56.6%	Doomscrolling Tendency Scale	OTHER	Quantitative dataset; not pooled
Doğan Laçin and Kiye (2025)	Türkiye/Middle East	Cross-sectional	Adults	348 (3 univariate outliers removed)	NR (18–50)	68.1%	DSS-15	PWB, ER	Meta-analysis
Kaya and Griffiths (2025)	Türkiye/Middle East	Cross-sectional	Adults	415	34.98	75.4%	DSS-4/short form	PD, OTHER	Meta-analysis
Canoğulları (2025)	Türkiye/Middle East	Cross-sectional	University students	460	NR (ages 18–30)	78%	DSS-15	PD, SMA	Meta-analysis
Hussain et al. (2025)	Pakistan/South Asia	Cross-sectional	Young adults	201	21.45	66.2%	DSS-15	BO, OTHER	Quantitative dataset; not pooled

This table describes the 30 reports with structured quantitative extraction; it does not imply that every report contributed to primary pooling. Twenty-one studies (23 independent analytic samples) contributed 40 primary effects, while 9 additional reports in this table contributed quantitative but nonprimary evidence. Together with 14 further narrative reports, 23 reports were outside the primary pools. Sample size refers to the analyzed report/sample level. DSS-15, 15-item Doomscrolling Scale; DSS-4, four-item Doomscrolling Scale; SMDS, Social Media Doomscrolling Scale; NR, not reported. Full information for all 44 reports is provided in Supplementary Table S1.

Most reports used cross-sectional, survey-based, correlational, or psychometric designs, with a smaller number of longitudinal or experimental units. The evidence base was geographically uneven, with Türkiye contributing the largest share. Other settings included China, India, Pakistan, Singapore, Italy, the United Kingdom, Canada, Iran, the United States, and international or multi-country samples.

Samples included university students, community adults, social media users, occupational groups, and crisis-affected populations. Recruitment, sample composition, age, and sex distribution varied considerably across reports. These differences, along with variation in instruments and design, were considered when interpreting heterogeneity and generalizability rather than being treated as interchangeable study characteristics.

Doomscrolling was assessed with full-length DSS-15 measures, DSS-4 versions, SMDS measures, and a small number of adapted or author-defined instruments. The analysis dataset contained 42 rows: 40 primary effects across the five prespecified outcome domains and two perceived-stress correlations retained for narrative synthesis.

### 3.3 Doomscrolling and outcome measures

Doomscrolling was assessed using several related but not identical measurement approaches (Figure 2A). The most common exposure measure was the 15-item Doomscrolling Scale or its translated and adapted versions (*n* = 14), followed by short-form four-item versions of the scale (*n* = 9) and the Social Media Doomscrolling Scale (*n* = 4). A smaller number of studies used an *ad hoc* author-defined multi-item measure, a single-item measure, or a measure classified as other or unclear. Although most studies were anchored in established doomscrolling instruments, the evidence base still showed heterogeneity in scale length, platform framing, and construct operationalization.

**Figure 2:**
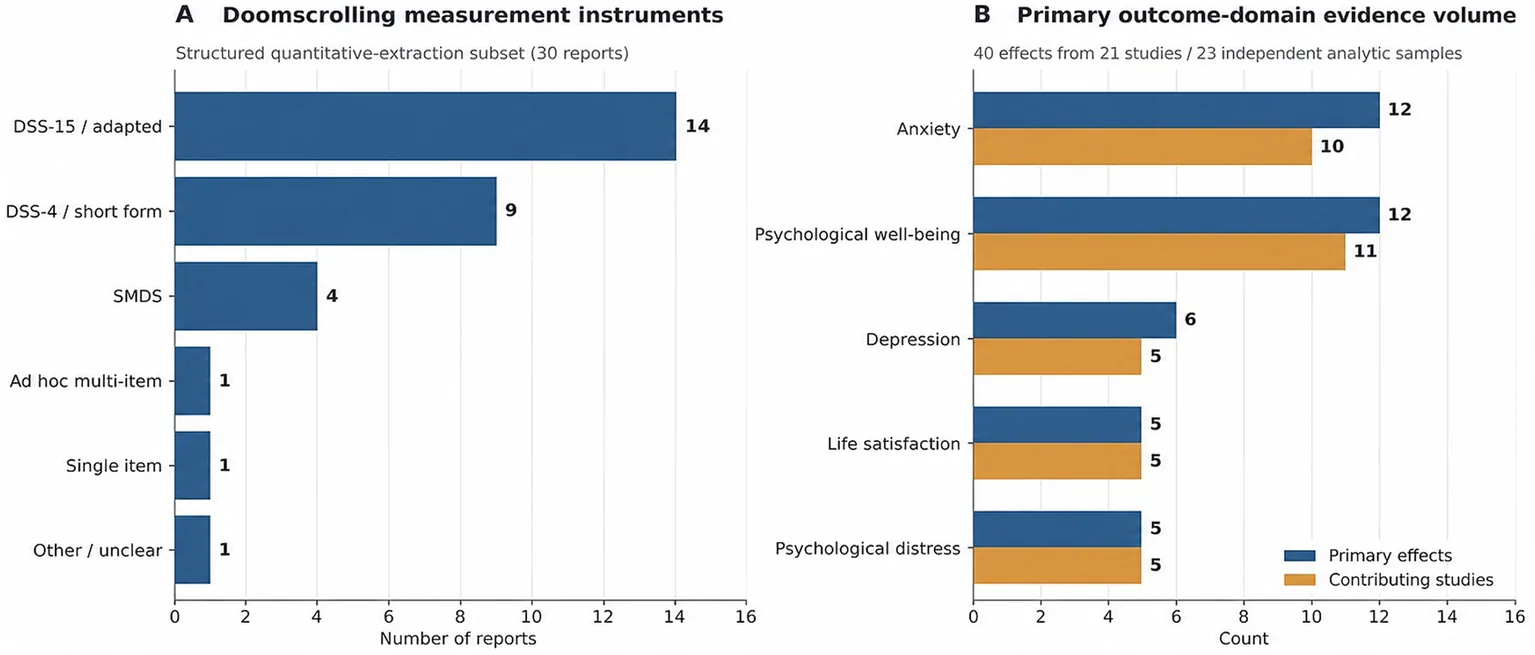
Doomscrolling measurement instruments and primary outcome-domain evidence volume. **(A)** Describes the 30-report structured quantitative-extraction subset (21 primary-meta studies plus 9 reports with nonprimary quantitative evidence). **(B)** Shows the 40 primary effects: anxiety *k* = 12, psychological well-being *k* = 12, depression *k* = 6, life satisfaction *k* = 5, and psychological distress *k* = 5. DSS-15, 15-item Doomscrolling Scale; DSS-4, four-item Doomscrolling Scale; SMDS, Social Media Doomscrolling Scale.

The five primary pools contained 40 effects: anxiety *k* = 12, psychological well-being *k* = 12, depression *k* = 6, life satisfaction *k* = 5, and psychological distress *k* = 5 (Figure 2B). Twenty-one studies contributed these effects through 23 independent analytic samples. Additional report-level or outcome-level quantitative evidence remained nonprimary when it was adjusted, duplicated, domain-specific, sparse, or otherwise outside the selection rules.

Outcome instruments also varied across domains. Anxiety was commonly measured using instruments such as the Generalized Anxiety Disorder Scale, the DASS-21 anxiety subscale, and future-oriented anxiety measures, including the Dark Future Scale. Depression was assessed using instruments including the PHQ-9, DASS-21 depression subscale, and Brief Symptom Inventory depression subscale. Psychological well-being and life satisfaction were commonly assessed using the Psychological Well-Being Scale, the Warwick–Edinburgh Mental Well-being Scale or its short form, and the Satisfaction with Life Scale. Psychological distress was mainly assessed using DASS-21 total scores or psychological-distress scales. Given this measurement heterogeneity, quantitative analyses were conducted separately by outcome domain rather than as a single pooled mental-health outcome.

### 3.4 Risk of bias

All 46 appraisal units were critically appraised. The review-derived overall distribution was 12 low risk, 28 moderate risk, 6 high risk, and 0 unclear. Among the 21 primary-meta appraisal units, 7 were low risk, 14 moderate risk, and none high risk. Among 25 narrative or nonprimary appraisal units, 5 were low risk, 14 moderate risk, and 6 high risk.

Item-level judgments remained primary. Overall categories were derived proportionally across the 8-, 11-, and 13-item JBI checklists and were not official JBI scores. Risk-of-bias ratings did not automatically exclude evidence; they informed sensitivity analyses and GRADE. Complete item-level judgments, checklist identities, and the review-derived algorithm are provided in Supplementary Table S2.

### 3.5 Primary meta-analyses

Random-effects meta-analyses were conducted for five outcome domains (Figure 3). Anxiety showed a positive association with doomscrolling (*k* = 12, *r* = 0.352, 95% CI 0.273–0.426; *I*^2^ = 90.0%; 95% prediction interval 0.076 to 0.578).

**Figure 3:**
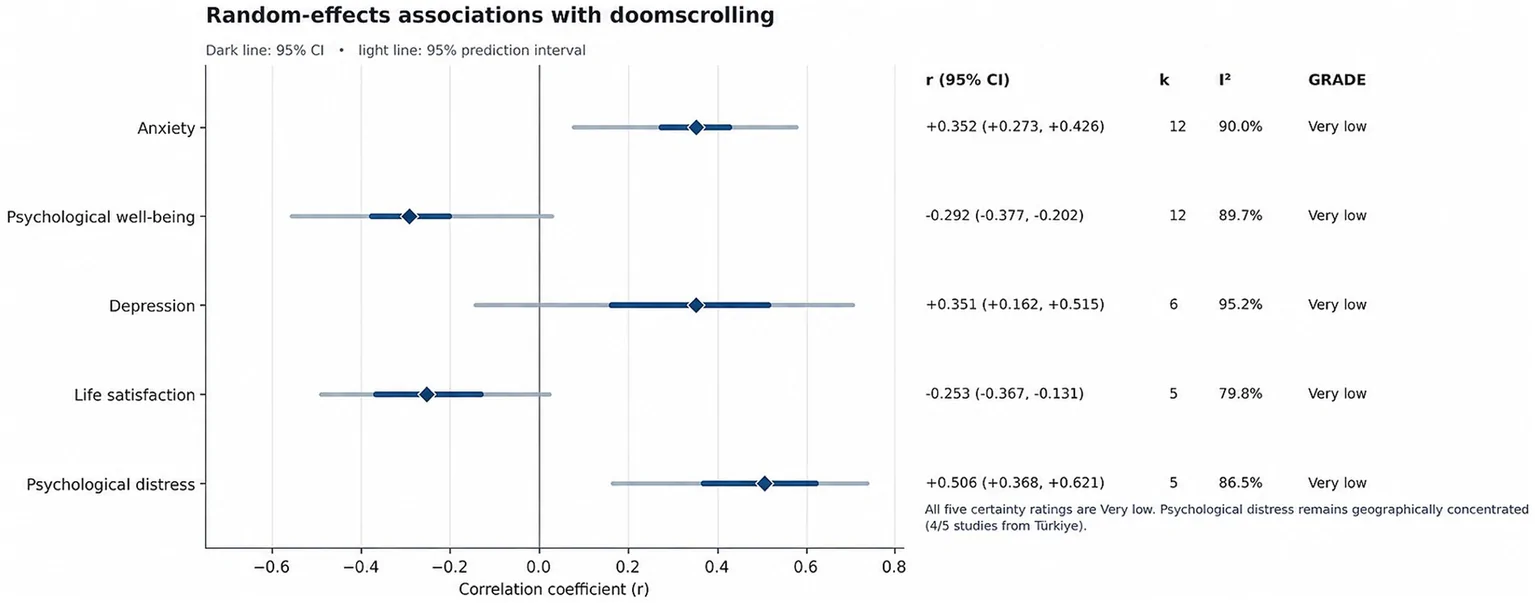
Summary of random-effects associations between doomscrolling and five primary psychological outcomes. Diamonds show pooled correlations presented on the *r* scale, dark lines 95% confidence intervals, and light lines 95% prediction intervals. Psychological distress: *K* = 5, *r =* 0.506 (95% CI 0.368–0.621), *I*^2^ = 86.5%, prediction interval 0.166–0.738. All five GRADE certainty ratings were very low. Across the figure, *k* denotes the number of prixmary effects; 40 effects arose from 21 studies and 23 independent analytic samples.

Psychological well-being showed a negative association (*k* = 12, *r* = −0.292, 95% CI − 0.377 to −0.202; *I*^2^ = 89.7%; prediction interval −0.557 to 0.027). Depression showed a positive association (*k* = 6, *r* = 0.351, 95% CI 0.162–0.515; *I*^2^ = 95.2%; prediction interval −0.143 to 0.705).

Life satisfaction showed a negative association (*k* = 5, *r* = −0.253, 95% CI − 0.367 to −0.131; *I*^2^ = 79.8%; prediction interval −0.491 to 0.021). The largest pooled association was observed for psychological distress (*k* = 5, *r* = 0.506, 95% CI 0.368 to 0.621; *I*^2^ = 86.5%; prediction interval 0.166–0.738). Heterogeneity was therefore high, and four of the five contributing studies were from Türkiye.

Directions were consistent with higher doomscrolling accompanying greater anxiety, depression, and psychological distress and lower psychological well-being and life satisfaction. However, high heterogeneity and broad prediction intervals in several domains indicate that the pooled estimates should not be treated as universal effects.

Study-level forest plots are provided in Supplementary Figure S1.

### 3.6 Sensitivity and publication bias analyses

Exploratory measure-type analyses compared DSS-15 with DSS-4/SMDS for anxiety and psychological well-being. For anxiety, DSS-15 yielded *k* = 6, *r* = 0.401 (95% CI 0.234 to 0.545; *I*^2^ = 94.9%), whereas DSS-4/SMDS yielded *k* = 6, *r* = 0.304 (95% CI 0.266 to 0.342; *I*^2^ = 0%); the subgroup test was not significant (*p* = 0.161). For psychological well-being, DSS-15 yielded *k* = 6, *r* = −0.312 (95% CI − 0.497 to −0.099; *I*^2^ = 94.4%), whereas DSS-4/SMDS yielded *k* = 6, *r* = −0.270 (95% CI − 0.329 to −0.209; *I*^2^ = 46.8%); the subgroup test was not significant (*p* = 0.661). Directions were consistent and heterogeneity differed descriptively, but no statistically supported subgroup difference was demonstrated.

Excluding Türk-Kurtça and Kocatürk (2025) [*N* = 443; trait anxiety] left 11 anxiety effects (*r* = 0.364, 95% CI 0.283 to 0.441; *I*^2^ = 89.7%; prediction interval 0.091 to 0.586). Removing Türk-Kurtça and Kocatürk (2025) [*N* = 443; trait anxiety] for the suspected-overlap analysis produced the same result. Alternately removing Türk-Kurtça and Kocatürk (2025) [*N* = 553; GAD-7] yielded *k* = 11, *r* = 0.353 (95% CI 0.265–0.435; *I*^2^ = 91.0%; prediction interval 0.054 to 0.594). These checks did not resolve the underlying overlap uncertainty but did not materially change the main anxiety estimate.

The low-risk-of-bias sensitivity for anxiety included five effects (*r* = 0.355, 95% CI 0.209 to 0.486; *I*^2^ = 86.7%; prediction interval 0.005 to 0.628). For psychological well-being, three low-risk effects yielded *r* = −0.192 (95% CI − 0.542 to 0.214; *I*^2^ = 92.7%). Low-risk evidence was too sparse for meaningful pooling for depression (*k* = 1), life satisfaction (*k* = 2), or psychological distress (*k* = 2); no pooled low-risk estimate was claimed for those domains.

Psychological distress remained geographically concentrated: four of five contributing studies (80%) were from Türkiye and one was the Italian Soraci study (*r* = 0.658; *N* = 300). The Türkiye-only sensitivity estimate was *r* = 0.463 (95% CI 0.382 to 0.537; *I*^2^ = 41.5%). This lower Türkiye-only heterogeneity is a valid sensitivity result, not the heterogeneity of the updated five-study primary pool.

Leave-one-out analyses did not change the direction of any primary association. The largest absolute change in a pooled correlation was observed for psychological distress (maximum |Δr| = 0.043), when the Soraci effect was removed. In analyses restricted to Pearson-based estimates, depression decreased from approximately *r* = 0.35 to *r* = 0.30; the directions of the primary associations were otherwise unchanged. Cluster-robust results were estimable for anxiety and psychological well-being and remained consistent with the corresponding main models. Small-study effects were assessed only for anxiety and psychological well-being. Egger’s tests were not significant for anxiety (*p* = 0.962) or psychological well-being (*p* = 0.301), and trim-and-fill imputed no studies in either domain (k0 = 0). Publication-bias tests were not conducted for depression, life satisfaction, or psychological distress because k was below 10.

### 3.7 Narrative synthesis of non-poolable outcomes

Twenty-three reports contributed narrative or nonprimary quantitative evidence. This group included reports without eligible zero-order correlations as well as reports containing adjusted, path-based, experimental, domain-specific, duplicated, or sparse quantitative evidence. The label therefore does not imply that every one of these reports was narrative-only.

Recent evidence included Soraci et al. (2025), Türk-Kurtça and Kocatürk (2025), and Dominguez-Rodriguez et al. (2025). Soraci contributed anxiety, depression, life satisfaction, and psychological well-being; one eligible DSS-15 psychological-distress correlation (*r* = 0.658) reported in its supplement was included once in the primary distress pool. Türk-Kurtça’s DSS-15 trait-anxiety correlation was included in the primary anxiety pool and examined in trait-exclusion and overlap sensitivities; intolerance of uncertainty and resilience remained nonprimary. Dominguez-Rodriguez reported adjusted psychological-outcome regressions and therefore remained narrative/nonprimary; zero-order correlations recomputed from deposited individual-level data were not eligible for primary analysis because pooling required article- or supplement-reported effects.

Problematic social media use and social media addiction were retained as adjacent correlates rather than substitute exposures. Similarly, climate-specific doomscrolling was discussed as a domain-specific form of negative-news engagement, not co-entered with a general doomscrolling measure in the same pool. For Yang et al., anxiety and depression correlations contributed where eligible, whereas the sleep/insomnia association remained narrative because scoring polarity could not be verified and was not reverse-coded by assumption (Yang et al., 2024).

### 3.8 Certainty of evidence

GRADE certainty was very low for anxiety, psychological well-being, depression, life satisfaction, and psychological distress (Supplementary Table S3). The cross-sectional observational evidence, risk-of-bias profile, inconsistency, imprecision, indirectness where relevant, small numbers of effects, and residual confounding precluded higher ratings.

Anxiety, psychological well-being, depression, and life satisfaction were rated very low because substantial inconsistency and, for several domains, prediction intervals crossing the null or sparse low-risk evidence limited confidence. The absence of detected funnel asymmetry for anxiety and psychological well-being did not offset these limitations.

Psychological distress was also rated very low. Although it had the largest pooled correlation, the evidence comprised only five effects, was predominantly cross-sectional, had high inconsistency (*I*^2^ = 86.5%), remained geographically concentrated, and was vulnerable to residual confounding. Publication bias could not be assessed because k < 10.

## 4 Discussion

### 4.1 Summary of main findings

Across 21 studies, 23 independent analytic samples, and 40 primary effects, doomscrolling was associated with greater anxiety, depression, and psychological distress and with lower psychological well-being and life satisfaction. The associations were outcome-specific and correlational; every GRADE rating was very low.

Psychological distress showed the largest pooled correlation, but this estimate was accompanied by high heterogeneity and remained geographically concentrated, with four of five contributing studies from Türkiye. The Italian Soraci sample broadens the evidence base but does not resolve the need for cross-cultural replication. No low-risk-only pooled estimate was supported for psychological distress because only two effects met the low-risk criterion.

The results should therefore be interpreted as patterns of association rather than evidence of causal direction or effect. Reverse directionality and shared influences—including negative affectivity, intolerance of uncertainty, general problematic digital engagement, crisis exposure, and socioeconomic or contextual factors—remain plausible.

### 4.2 Comparison with existing reviews and contribution

This review adds outcome-specific pooled estimates to earlier narrative and scoping syntheses (Güme, 2024; Sharpe et al., 2026). Recent empirical reports extend the evidence base across validation, trait-anxiety, and domain-specific negative-news contexts (Soraci et al., 2025; Türk-Kurtça and Kocatürk, 2025; Dominguez-Rodriguez et al., 2025). Soraci et al. contributed multi-domain validation evidence, including one eligible supplement-reported psychological-distress correlation. Türk-Kurtça and Kocatürk contributed trait-anxiety evidence and related uncertainty and resilience findings, whereas Dominguez-Rodriguez et al. contributed adjusted evidence concerning general and climate-specific doomscrolling.

The synthesis also sharpens construct boundaries. Doomscrolling combines predominantly negative or threatening content with persistent or repetitive engagement and difficulty disengaging. Problematic social media use primarily concerns dysregulated platform use; general negative-news exposure need not involve compulsive scrolling; and problematic internet use is broader and content-nonspecific. Dominguez-Rodriguez’s comparison of general and climate-specific doomscrolling illustrates overlap without establishing equivalence (Dominguez-Rodriguez et al., 2025).

These distinctions do not imply that digital behaviors are independent. Doomscrolling may coexist with problematic social media use, habitual news checking, cyberchondria, or other repetitive online behaviors. The value of a specific synthesis is to avoid attributing the psychological correlates of one exposure to all screen-based behavior and to define which exposure should be measured in future causal research.

### 4.3 Possible psychological mechanisms

The pattern is compatible with perseverative negative-affect engagement. Repeated contact with adverse information can accompany rumination, sustained negative affect, and difficulty disengaging; those processes map most directly onto depression and broad psychological distress. The evidence is correlational, so this mapping identifies hypotheses rather than demonstrated mediators.

Threat monitoring and intolerance of uncertainty are particularly relevant to anxiety. Individuals who experience uncertainty as aversive may repeatedly seek updates in an attempt to gain orientation or control, while each new negative item can renew vigilance (Türk-Kurtça and Kocatürk, 2025; Kaya and Griffiths, 2025). Trait anxiety may precede scrolling, scrolling may coincide with symptom escalation, or both may reflect shared dispositions; the trait-anxiety exclusion sensitivity showed that the pooled anxiety association did not depend on the Türk-Kurtça trait measure.

A psychological-resource perspective complements symptom-focused accounts. Repetitive attention to distressing content may coincide with lower perceived control, reduced opportunities for restorative activity, and poorer broader functioning, providing a conceptual link to psychological well-being and life satisfaction. Psychological resilience in Türk-Kurtça and Kocatürk and coping-related constructs in Dominguez-Rodriguez illustrate this resource-oriented evidence, while remaining correlational or nonprimary (Türk-Kurtça and Kocatürk, 2025; Dominguez-Rodriguez et al., 2025).

Together, threat/uncertainty, negative-affect/perseverative, and resource/well-being processes provide an outcome-specific framework for future tests. Longitudinal temporal models, ecological momentary assessment, and experiments are required to determine ordering and mechanism.

### 4.4 Heterogeneity and geographic concentration

Heterogeneity was high in all five primary domains. Plausible contributors include full-length versus short or platform-specific doomscrolling instruments, variation in psychological outcome measures, geographic concentration, student versus community or occupational samples, cross-sectional design, trait versus symptom-based anxiety, and residual confounding. These features may shape the distribution of estimates, but the analyses did not identify a definitive source of heterogeneity.

Geographic concentration was especially important for psychological distress. Four of five effects were from Türkiye and one was from Italy. The pooled estimate was *r* = 0.506 (95% CI 0.368–0.621; *I*^2^ = 86.5%), whereas the Türkiye-only sensitivity was *r* = 0.463 (95% CI 0.382–0.537; *I*^2^ = 41.5%). The contrast should not be interpreted as a causal country effect; it shows that the Italian effect broadened coverage while increasing between-study dispersion.

Exploratory measure-type analyses showed directions that were consistent for anxiety and psychological well-being. Heterogeneity differed descriptively between DSS-15 and DSS-4/SMDS groups, but neither between-group test was significant (anxiety *p* = 0.161; psychological well-being *p* = 0.661). Measure type therefore cannot be said to explain heterogeneity.

Further variation arose from sample composition, crisis contexts, outcome-instrument choice, and incomplete handling of confounding. Standardized exposure definitions and coordinated measurement would improve comparability, but replication across languages, age groups, countries, and information environments remains necessary.

### 4.5 Strengths and limitations

Strengths include broad multi-source searching, explicit construct boundaries, outcome-specific random-effects models with prediction intervals, complete appraisal of all 46 units using design-appropriate JBI tools, and transparent separation of primary from narrative, adjusted, domain-specific, duplicated, and public-data-derived evidence. Additional exploratory analyses examined measure type, trait anxiety, suspected overlap, low risk of bias, and geographic concentration without reclassifying them as prespecified primary analyses.

The evidence was dominated by cross-sectional and correlational designs. Accordingly, the associations cannot determine whether doomscrolling precedes poorer mental health, follows existing distress, or reflects shared vulnerabilities. Residual confounding is likely because baseline mental health, personality, general digital-media use, socioeconomic conditions, crisis exposure, and other contextual variables were inconsistently measured or controlled.

Generalizability is limited by geographic and sample concentration. Psychological distress remained especially constrained: four of five effects came from Türkiye, and the only non-Türkiye effect came from one Italian validation sample. High heterogeneity in the five-effect pool reinforces the need for independent cross-cultural replication rather than a claim of stability.

Measurement heterogeneity also limits inference. Full-length, short-form, platform-specific, translated, and author-defined doomscrolling instruments were combined only when they met the same exposure and selection rules, while outcome instruments varied within domains. Twenty-three reports remained outside primary pooling because their evidence was adjusted, nonprimary, duplicated, domain-specific, sparse, experimental, or otherwise non-comparable. Publication bias could be examined only for anxiety and psychological well-being.

### 4.6 Implications and future directions

Future studies should use longitudinal designs, ecological momentary assessment, and experiments to establish temporal ordering and test threat, uncertainty, rumination, negative affect, and resource pathways. Cross-cultural validation should extend beyond the currently concentrated settings, and preregistered causal or intervention studies should specify exposure definition, primary outcomes, confounders, and analytic decisions in advance.

Measurement standardization is equally important. Studies should report the exact doomscrolling scale and scoring direction, distinguish general from domain-specific negative-news engagement, avoid treating problematic social media use as an interchangeable exposure, and routinely report zero-order correlations alongside adjusted models. Direct psychometric comparisons of DSS-15, DSS-4, SMDS, and domain-specific measures would clarify common and distinct content.

Practical recommendations must remain tentative. The Perspective by Shabahang and Weber proposes mindful and harmonious consumption of negative news—more deliberate, agentic, and bounded engagement rather than complete avoidance (Shabahang and Weber, 2025). This is a useful conceptual direction for future intervention development, not evidence of intervention effectiveness. Candidate strategies should be tested in preregistered trials and may include attention to goals, emotional responses, stopping cues, information sufficiency, and balance between staying informed and preserving psychological functioning.

## 5 Conclusion

Doomscrolling was associated with greater anxiety, depression, and psychological distress and with lower psychological well-being and life satisfaction. Psychological distress had the largest pooled correlation, but high heterogeneity and concentration in Türkiye limit its generalizability.

All five outcome domains were rated very low certainty. The findings identify reproducible correlational patterns, not causal effects or established clinical targets. Stronger inference requires standardized measures, transparent zero-order reporting, longitudinal and ecological momentary assessment designs, experiments, and preregistered cross-cultural replication.

## Data Availability

The original contributions presented in the study are included in the article/Supplementary material, further inquiries can be directed to the corresponding author.
