# Supplementary material for: Doomscrolling, mental health, and psychological well-being in adolescents and adults: a systematic review and meta-analysis
Source: Front Psychol. 2026 Sep 18;17:1893800. doi: 10.3389/fpsyg.2026.1893800 (PMC13630949; doi:10.3389/fpsyg.2026.1893800)
Supplement: Supplementary file 1 [file Data_Sheet_1.PDF]

Supplementary Materials

Doomscrolling, Mental Health, and Psychological Well-Being in Adolescents and Adults: A Systematic Review and Meta-Analysis

PROSPERO CRD420261395295

Includes Supplementary Methods 1–2, Supplementary Tables S1–S4, and Supplementary Figure S1.

Supplementary Methods 1. Full search strategy

All searches were conducted on 20 May 2026. No language restriction was applied. No year, study design, or publication status restrictions were applied at the retrieval stage; the eligibility window was applied at the screening stage. All search strings used free-text terms in title, abstract, and, where supported, keyword fields. No controlled vocabulary terms (MeSH or Emtree) were applied, given the recent emergence of the doomscrolling construct and its inconsistent indexing in bibliographic databases.

For PubMed, Web of Science, and Scopus, the doomscrolling exposure block was combined with a broad outcome block to enhance precision. For Embase, the Cochrane Library, CNKI, and ClinicalTrials.gov, the doomscrolling exposure block was used alone to maximize sensitivity, given the smaller indexed volume of these sources and the recognition that some eligible studies may discuss mental health outcomes only in the full text rather than in the abstract.

S1.1 PubMed

Search date: 20 May 2026 Interface: PubMed.gov Field codes: [Title/Abstract] Limits applied: None Records retrieved: 32

(doomscrolling[Title/Abstract] OR doom-scrolling[Title/Abstract] OR "doom scrolling"[Title/Abstract] OR doomsurfing[Title/Abstract] OR doom-surfing[Title/Abstract] OR "doom surfing"[Title/Abstract])

AND

("mental health"[Title/Abstract] OR depression[Title/Abstract] OR depressive[Title/Abstract] OR anxiety[Title/Abstract] OR stress[Title/Abstract] OR distress[Title/Abstract] OR wellbeing[Title/Abstract] OR well-being[Title/Abstract] OR "psychological well-being"[Title/Abstract] OR "life satisfaction"[Title/Abstract] OR loneliness[Title/Abstract] OR sleep[Title/Abstract] OR insomnia[Title/Abstract] OR trauma[Title/Abstract] OR "secondary traumatic stress"[Title/Abstract] OR PTSD[Title/Abstract] OR burnout[Title/Abstract])

S1.2 Embase

Search date: 20 May 2026 Interface: Embase.com Field codes: :ti,ab (title, abstract) Limits applied: None Records retrieved: 28

Note: The Embase search used the doomscrolling exposure block alone, without an outcome filter, to maximize sensitivity. This decision reflected the highly specific nature of the doomscrolling construct, which carries minimal risk of false-positive retrieval, and the recognition that some eligible studies may not include mental health terms within abstract-indexed fields. The full screening process subsequently applied the outcome eligibility criteria.

(doomscrolling:ti,ab OR 'doom-scrolling':ti,ab OR 'doom scrolling':ti,ab OR doomsurfing:ti,ab OR 'doom-surfing':ti,ab OR 'doom surfing':ti,ab)

S1.3 Web of Science Core Collection

Search date: 20 May 2026 Interface: Web of Science (Clarivate) Indexes searched: Science Citation Index Expanded (SCI-EXPANDED); Social Sciences Citation Index (SSCI); Arts & Humanities Citation Index (A&HCI); Emerging Sources Citation Index (ESCI) Field codes: TS= (Topic: title, abstract, author keywords, Keywords Plus) Limits applied: None Records retrieved: 57

TS=(doomscrolling OR doom-scrolling OR "doom scrolling" OR doomsurfing OR doom-surfing OR "doom surfing")

AND

TS=("mental health" OR depression OR depressive OR anxiety OR stress OR distress OR wellbeing OR "well-being" OR "psychological well-being" OR "life satisfaction" OR loneliness OR sleep OR insomnia OR trauma OR "secondary traumatic stress" OR PTSD OR burnout)

S1.4 Cochrane Library (CENTRAL)

Search date: 20 May 2026 Interface: Cochrane Library (Wiley) Database within Cochrane Library: CENTRAL Field codes: All fields (default) Limits applied: None Records retrieved: 1

Note: The Cochrane CENTRAL search used the doomscrolling exposure block alone, consistent with the rationale described for Embase.

doomscrolling OR doom-scrolling OR "doom scrolling" OR doomsurfing OR doom-surfing OR "doom surfing"

S1.5 Scopus

Search date: 20 May 2026 Interface: Scopus.com Field codes: TITLE-ABS-KEY (title, abstract, keywords) Limits applied: None Records retrieved: 69

TITLE-ABS-KEY (doomscrolling OR doom-scrolling OR "doom scrolling" OR doomsurfing OR doom-surfing OR "doom surfing")

AND

TITLE-ABS-KEY ("mental health" OR depression OR depressive OR anxiety OR stress OR distress OR wellbeing OR "well-being" OR "psychological well-being" OR "life satisfaction" OR loneliness OR sleep OR insomnia OR trauma OR "secondary traumatic stress" OR PTSD OR burnout)

S1.6 China National Knowledge Infrastructure (CNKI)

Search date: 20 May 2026 Interface: CNKI (cnki.net) Field codes: SU = (Subject: title, abstract, keywords combined) Limits applied: None Records retrieved: 2

Note: The CNKI search combined English orthographic variants with five candidate Chinese-language translations of the doomscrolling construct. As a standardized Chinese-language equivalent has not yet been

established, multiple translation variants were searched to maximize sensitivity to emerging Chinese-language literature.

SU = (doomscrolling OR "doom scrolling" OR doomsurfing OR "doom surfing" OR 末日刷屏 OR 末日滚动 OR 灾难刷屏 OR 负面新闻刷屏 OR 负性新闻刷屏)

English translation of Chinese-language terms (for the reader): 末日刷屏 (mò rì shuā píng) — "doomsday scrolling" 末日滚动 (mò rì gǔn dòng) — "doomsday scrolling / rolling" 灾难刷屏 (zāi nàn shuā píng) — "disaster scrolling" 负面新闻刷屏 (fù miàn xīn wén shuā píng) — "negative-news scrolling" 负性新闻刷屏 (fù xìng xīn wén shuā píng) — "negative-news scrolling"

S1.7 ClinicalTrials.gov

Search date: 20 May 2026 Interface: clinicaltrials.gov Field: All fields (Other Terms) Limits applied: None Records retrieved: 1

doomscrolling OR doom scrolling OR doomsurfing OR doom surfing

S1.8 Search summary table

Database / Platform	Search date	Records retrieved
PubMed (NLM)	20 May 2026	32
Embase (Elsevier)	20 May 2026	28
Web of Science Core Collection (Clarivate)	20 May 2026	57
Cochrane Library, CENTRAL (Wiley)	20 May 2026	1
Scopus (Elsevier)	20 May 2026	69
CNKI (CNKI Group)	20 May 2026	2
ClinicalTrials.gov (NLM)	20 May 2026	1

Records retrieved from databases: 189; records retrieved from registers: 1; total: 190

S1.9 Supplementary searches

Google Scholar (grey literature): searched on 20 May 2026 using the query doomscrolling OR "doom scrolling" OR doomsurfing OR "doom surfing". The first 200 results sorted by relevance were reviewed. Of 200 records identified, 131 were removed before screening (78 matches to the main databases and 53 cross-query duplicates), leaving 69 unique records screened. Forty-nine records were not advanced after supplementary title/snippet screening (24 title/snippet exclusions and 25 other records not advanced after title/snippet screening). Twenty

reports were sought for retrieval; 14 were not retrieved. Six reports were assessed for eligibility and all six were eligible.

Citation chasing: reference lists of all studies meeting full-text eligibility were hand-searched (backward citation chasing). Forward citations of Sharma, Lee and Johnson (2022), the original study introducing the Doomscrolling Scale, were screened in Google Scholar. No additional eligible records were identified through citation chasing.

Supplementary Methods 2. Effect size extraction and transformation rules

This document describes the rules used to extract effect sizes from individual studies and to transform them for quantitative synthesis. These rules were specified in the registered protocol before quantitative synthesis and applied uniformly across all eligible studies.

S2.1 Primary effect size metric

The primary effect size for the meta-analysis was the zero-order Pearson product-moment correlation coefficient (r) between doomscrolling and each eligible psychological outcome. The zero-order correlation was selected because (i) the included literature reports doomscrolling–outcome associations predominantly as bivariate correlations; (ii) zero-order correlations are not conditioned on additional covariates and therefore reflect a consistent estimand across studies that differ in their multivariable modeling approaches; and (iii) zero-order correlations can be transformed into a stabilized metric (Fisher's z) suitable for random-effects meta-analysis. Adjusted associations from multivariable regression models were not eligible as the primary effect size because the set of adjustment covariates varied substantially across studies.

S2.2 Direction of correlations

Correlations were extracted in their reported direction, with the following interpretive convention:

A positive correlation indicates that higher doomscrolling scores are associated with higher scores on the outcome instrument.

A negative correlation indicates that higher doomscrolling scores are associated with lower scores on the outcome instrument.

For adverse psychological outcomes (depression, anxiety, perceived stress, psychological distress, post-traumatic stress symptoms, secondary traumatic stress, burnout, loneliness, insomnia, negative affect), a positive correlation therefore indicates greater symptom burden or greater dysfunction. For adaptive psychological outcomes (psychological well-being, life satisfaction, positive affect, self-esteem), a negative correlation indicates lower well-being or lower adaptive functioning. The directional interpretation of each correlation was verified against the scoring instructions of the original outcome instrument as reported in each study; correlations from instruments with reverse-scored items but uninterpretable total-score direction were retained for narrative synthesis only.

S2.3 Spearman rank-order correlations

When Spearman's rank-order correlation coefficient (ρ) was the only available zero-order association between doomscrolling and an eligible outcome, ρ was treated as an approximation of the corresponding Pearson correlation for quantitative synthesis. A completed sensitivity analysis excluded all Spearman-based estimates from each primary outcome-domain meta-analysis. Studies reporting both Pearson's r and Spearman's ρ were represented by the Pearson coefficient.

S2.4 Fisher's z transformation

For quantitative synthesis, each correlation coefficient was transformed to Fisher's z using the standard formula:

$$z = 0.5 \times \ln[(1 + r) / (1 - r)]$$

The sampling variance of Fisher's z was calculated as:

$$Var(z) = 1 / (n - 3)$$

where n is the sample size contributing to the correlation estimate. The standard error of Fisher's z was calculated as the square root of the variance:

$$SE(z) = 1 / \sqrt{(n - 3)}$$

Pooled Fisher's z estimates were back-transformed to Pearson's r for presentation and interpretation using the inverse formula:

$$r = (e^{2z} - 1) / (e^{2z} + 1)$$

Pooled confidence intervals and prediction intervals were calculated on the Fisher's z scale and back-transformed to the correlation scale by applying the inverse transformation to each interval bound.

S2.5 Derivation of standard errors and confidence intervals when not directly reported

When a study reported a correlation coefficient and sample size but did not report a 95% confidence interval or standard error, the standard error was derived from the Fisher's z variance formula using the reported sample size. When a study reported a correlation coefficient but did not clearly specify the sample size contributing to the correlation, the most conservative (smallest relevant) analytical sample size was used to derive Fisher's z variance. These derivations were documented in the data extraction file at the study–effect level.

S2.6 Effect sizes not treated as zero-order correlations

The following effect-size types were not treated as equivalent to zero-order correlations and were not converted into Pearson's r for inclusion in the primary meta-analysis:

Standardized regression coefficients (β) from multivariable linear regression or structural equation models. These coefficients are conditioned on the specific set of covariates in each model and therefore do not estimate the same population parameter as a zero-order correlation. Conversion formulas based on β alone (e.g., Peterson and Brown, 2005) yield approximate r values that may differ from the true zero-order r by an unpredictable margin.

Unstandardized regression coefficients (b) from regression or multilevel models. These coefficients are expressed in the original outcome scale and depend on the variance of the predictor and outcome variables, making direct conversion to a correlation coefficient inadvisable without additional information on the variance structure.

Mediation path coefficients (path a, path b, indirect effect). These coefficients describe components of a hypothesized causal pathway rather than a marginal bivariate association.

Multilevel model estimates (γ), including coefficients from daily diary, experience-sampling, and longitudinal multilevel studies. These estimates describe within-person or between-person associations under specific multilevel parameterizations and are not directly comparable to between-person zero-order correlations.

Experimental effect sizes from between-group comparisons (Cohen's d, standardized mean differences). These effect sizes describe the magnitude of a manipulated group difference rather than a bivariate correlation in a naturalistic sample.

Outcomes with ambiguous scoring direction or reverse-scored composite scales where the directional interpretation of the reported correlation could not be confirmed from the study text.

Studies reporting only these effect-size types or providing only correlations of indeterminate direction were retained for narrative synthesis as described in Methods Section 2.11.

S2.7 Multiple effect sizes within the same study

When a study reported more than one eligible effect size contributing to the same outcome domain (for example, multiple outcome instruments measuring depression, multiple subscales of a single instrument, or correlations reported separately for subgroups within the same sample), the following selection rules were applied to identify a single representative effect for the primary analysis:

The most widely used and best-validated measure for the outcome domain was prioritized.

Total-score correlations were prioritized over subscale-score correlations when both were reported.

Whole-sample correlations were prioritized over subgroup-specific correlations.

Alternative eligible effect sizes from the same study and outcome domain were retained in the extraction file and considered for sensitivity or narrative assessment.

When a study reported effect sizes across distinct outcome domains, each effect was extracted as a separate study–outcome row and contributed to the meta-analysis of its respective outcome domain.

S2.8 Effect-size handling for non-poolable studies

Studies that met the conceptual eligibility criteria but did not provide an extractable zero-order correlation in any of the formats described in Sections S2.1 to S2.5 were retained for narrative synthesis. Their reported effect estimates, whether standardized coefficients, mediation paths, multilevel parameters, or experimental effects, were summarized descriptively in the narrative synthesis with reference to the population, doomscrolling measure, outcome construct, and direction of association.

Supplementary Table S1. Data extraction table

Project: Doomscrolling, Mental Health, and Psychological Well-Being in Adolescents and Adults: A Systematic Review and Meta-Analysis (PROSPERO CRD420261395295).

The final review contains 44 reports. The structured quantitative-extraction subset contains 30 reports: 21 primary-meta studies and 9 reports with quantitative but nonprimary evidence. Fourteen additional reports were retained narratively and are listed in Supplementary Table S1c. Inclusion in the primary meta-analysis was determined at the study–outcome level.

Supplementary Table S1a. Study-level characteristics of the 30-report structured quantitative-extraction subset

Note. These 30 reports comprise the structured quantitative-extraction subset and should not be interpreted as the total number of reports in the systematic review. Twenty-one studies contributed at least one primary effect; nine reports contributed quantitative but nonprimary evidence. NR = not reported.

First author	Year	Country / region	Study design	Population	Recruitment setting	Sample size	Mean age	Age range	Female (%)	Doomscrolling measure	Included in primary meta	Narrative/partial status	Notes
Ulaş E	2026	Türkiye	Cross-sectional	University students	Earthquake-affected universities (Kahramanmaraş)	414	NR	NR (undergraduate)	47.8%	Doomscrolling Scale (Sharma 2022; Turkish adaptation)	No	Quantitative/nonprimary evidence	Sample from 2023 Kahramanmaraş earthquake-affected universities
Camadan F	2025	Türkiye	Cross-sectional descriptive-correlational	General adults	Online (social media)	663	25.00	18-77	80.1%	Doomscrolling Scale - Short Form (DS-SF) (Sharma 2022 4-item short / Turkish: Satici 2023)	No	Whole-study narrative-only (only path b reported)	Online convenience sample of Turkish adults
Ang CS	2025	Singapore	Cross-sectional	Social media users	Convenience sample via social media	235	29.81	NR	76.2%	Social Media Doomscrolling Scale (SMDS) (Shabahang 2022)	Yes	—	Convenience sample, Singapore
Yıldırım U	2025	Türkiye	Cross-sectional	Other (maritime deck cadets)	Onboard maritime training programmes	380	NR	NR (cadet students)	8.9%	Doomscrolling Scale - Short Form (Sharma 2022 / Turkish: Satici 2023)	Yes	—	Male-dominated cadet sample (91.1% male)
Dominguez-Rodriguez A	2025	Netherlands / international online sample	Cross-sectional	Mixed adult sample	Online international sample (climate change focus)	365	25.12	18-85	55%	Doomscrolling Scale + Climate-Change-Specific Doomscrolling Scale (CCDS) (Sharma 2022 DSS + author-developed CCDS)	No	Whole-study narrative-only (only adjusted regression b reported)	Mixed undergraduate/general public; mean DS score = 2.37 (SD = 1.01)

First author	Year	Country / region	Study design	Population	Recruitment setting	Sample size	Mean age	Age range	Female (%)	Doomscrolling measure	Included in primary meta	Narrative/partial status	Notes
Yang L	2024	China	Two-study cross-sectional (Study 1 validation N=2885; Study 2 mediation N=578)	University students	Chinese university student survey	Study 1 2885; Study 2 578	19.36 (Study 1); 19.41 (Study 2)	18-23	39.41 % (Study 1%); 37.54 % (Study 2%)	Chinese Doomscrolling Scale (DS-15) + short DS-4 (Sharma 2022; Yang Chinese translation)	Partial	Partial — Depression and Anxiety included; Insomnia retained for narrative synthesis because scoring direction could not be verified from the report or supplement	79.58% freshmen; 70.92% from rural areas
McCutcheon LE	2024	Iran; United States	Cross-sectional cross-cultural	University students	University recruitment in Iran and US	812	Iran 25.31; US 20.04	NR	Iran 79%; US 68%	Social Media Doomscrolling Scale (SMDS) (Shabahang 2022)	Yes	—	Separate analyses by country recommended due to cultural difference
Taskin S	2024	Türkiye	Cross-sectional	Social media users	Online Turkish social media user sample	400	NR	NR	NR	Doomscrolling Scale (Turkish adaptation) (Sharma 2022; Turkish: Satici 2023)	Yes	—	Turkish adult social media users; serial mediation context
Kartol A	2025	Türkiye	Cross-sectional, two-phase psychometric validation	General adults	Online survey across Turkish provinces (post-earthquake)	508 (Phase 2 with doomscrolling and outcomes)	NR	NR	NR	Doomscrolling Scale (Turkish) (Sharma 2022; Turkish: Satici 2023)	No	Whole-study narrative-only (only mediation β reported)	Post-earthquake sample; phase 2 contains doomscrolling measure
Kartol A	2023	Türkiye	Cross-sectional	General adults	Turkish adult earthquake survivors	402	NR (young adult majority)	NR	>70%	Doomscrolling Scale (Turkish) (Sharma 2022; Turkish: Satici 2023)	Yes	—	8% lost family member; ~25% had home destroyed; 20% still in tents/containers
Satıcı SA	2023	Türkiye	Multi-study cross-sectional (Study II N=419; Study III N=460)	Social media users	Online Turkish adult sample (23-81 provinces)	Study II 419; Study III 460	Study II 24.47; Study III 23.68	Study II 18-64; Study III 18-55	Study II 74%; Study III 74.6%	Doomscrolling Scale (Turkish validation, 15-item + 4-item tested) (Sharma 2022; Turkish: Satici 2023 (this study))	Yes	—	Study II = scale-personality correlations; Study III = scale-wellbeing-distress correlations
Buchanan K	2021	United Kingdom	Experimental (two between-group	Mixed adult	UK undergraduate +	Study 1 299; Study	Study 1 29;	NR	Study 1%	Experimental manipulation	No	Whole-study narrative-only	Experimental — between-group

First author	Year	Country / region	Study design	Population	Recruitment setting	Sample size	Mean age	Age range	Female (%)	Doomscrolling measure	Included in primary meta	Narrative/partial status	Notes
			experiments)	sample	YouTube-recruited general sample	2 603	Study 2 26		74%; Study 2% 59%	(Twitter feed @PandemicCovid20; YouTube reaction video) (Author-developed experimental exposure)		(experimental Cohen's d, not poolable with correlational meta)	COVID/kindness/control exposure
Antonysamy S	2026	India	Cross-sectional	University students	Indian college (Tamil Nadu)	399	20.5 (range)	18-24	NR	Doomscrolling Scale (Sharma 2022)	Yes	—	Indian undergraduate sample
Kaya B	2025	Türkiye	Cross-sectional	University students	Turkish university students online	496	20.26	NR	74.2%	Doomscrolling Scale - Short Form (DS-SF) (Sharma 2022 4-item / Turkish: Satici 2023)	Yes	—	Distinct from REC00046 (different Kaya paper, N=432)
Soraci P	2025	Italy	Cross-sectional psychometric validation	General adults	Italian adult sample	300	NR	NR	NR	DSS-15 primary associations; DSS-4 psychometrically evaluated, but no criterion-correlation matrix suitable for the primary outcome pools was reported.	Yes	Primary meta-analysis	Italian DSS validation paper; Spearman rho for main-article associations and Pearson r for the Supplementary Table S5 observed composite
Blacha S	2024	Canada	Cross-sectional, preregistered	University students	York University students	677	19.57	17+ undergraduate	72.2%	Doomscrolling Scale (Sharma 2022)	Yes	—	Diverse Canadian sample; 21% S Asian, 16.4% sexual minority
Kaya B	2025	Türkiye/international online	Cross-sectional	General adults	Online Turkish adults across 53/81 provinces	432	33.31	18-55	70.6%	Doomscrolling Scale - Short Form (Sharma 2022 4-item / Turkish: Satici 2023)	Yes	—	69% medium SES
Kurtça TT	2025	Türkiye	Cross-sectional path analysis	General adults	Turkish adult sample	553	NR	NR	NR	Doomscrolling Scale (Sharma 2022)	Yes	—	GAD-7, SMA, doomscrolling, cyberchondria —

First author	Year	Country / region	Study design	Population	Recruitment setting	Sample size	Mean age	Age range	Female (%)	Doomscrolling measure	Included in primary meta	Narrative/partial status	Notes
Shabahang R	2023	Iran	Cross-sectional online survey + SMDS development	Social media users	Iranian online shopping platforms	707 (40 outliers removed)	28.38	17-60	82.2%	Social Media Doomscrolling Scale (SMdoomS) (Shabahang 2022 - developed in this study)	Yes	—	Path model<br>Contains some 17-year-olds; mean age 28.38 — retained under adult/late-adolescent rule
Körün AB	2025	Türkiye	Cross-sectional SEM with network analysis	General adults	Turkish adult sample	811	23.01	NR	NR	Doomscrolling Scale (Sharma 2022 / Turkish: Satici 2023)	No	Whole-study narrative-only (only standardized SEM β coefficients available)	Externality of happiness, schadenfreude, family functioning, and mental well-being model; the available report did not permit reliable extraction of a directly comparable zero-order correlation, so the study was retained as nonprimary evidence.
Shabahang R	2024	Iran; United States	Cross-sectional SEM (cross-cultural)	University students	Iran + US convenience samples	Iran 620; US 180	Iran 26.75; US 20.06	NR	Iran 26.6%; US 75.0%	Social Media Doomscrolling Scale (SMDS) (Shabahang 2022)	Yes	—	Cross-cultural; separate effects per country recommended
Kütük H	2025	Türkiye	Cross-sectional SEM	University students	Turkish college students; convenience/snowball	406	22.31	18-52	82.3%	Doomscrolling Scale - Brief Form (TDS-BF) (Sharma 2022; Turkish: Satici 2023)	Yes	—	Istanbul-based; convenience/snowball sampling
Sharma B	2022	United States (OECD online panel)	Mixed-methods 3-study (Study 1 qualitative; Study 2 N=401 quantitative; Study 3 N=502	Social media users	OECD online panel	Study 2 401; Study 3 502	NR	NR	NR	Doomscrolling Scale (development paper) (Sharma 2022 - original)	Yes	—	Doomscrolling Scale development paper; Study 3 has criterion/convergent validity correlations

First author	Year	Country / region	Study design	Population	Recruitment setting	Sample size	Mean age	Age range	Female (%)	Doomscrolling measure	Included in primary meta	Narrative/partial status	Notes
			quantitative validation)										
Hughes IM	2024	United Kingdom	Two-study paper (Study 1 cross-sectional N=352; Study 2 daily-diary ESM N=126 / 630 obs)	Employees	UK employed adults via online panel	Study 1: 352; Study 2: 126 participants / 630 observations	NR	NR	NR	Work Doomscrolling (single item, author-developed) (Hughes 2024 - study-specific)	No	Whole-study narrative-only (single-item exposure + multilevel γ, not poolable)	Study 2 has within-person daily diary design; multilevel coefficients only
Türk-Kurtça T	2025	Türkiye	Cross-sectional SEM	General adults	Turkish adults via Google Forms	443	23.49	18-55	79.9%	Doomscrolling Scale (Sharma 2022 / Turkish: Satici 2023)	Yes	—	Online Google Forms recruitment
Biricik ZD	2026	Türkiye	Cross-sectional	University students	Communication faculty undergraduates, 7 Turkish regions	779	NR (17-55 + age bands)	17+ (mostly undergrad)	56.6%	Doomscrolling Tendency Scale (Turkish) (Bright & Logan 2022; Turkish: Satici 2022)	No	All effects are secondary / non-target outcomes (nomophobia + digital literacy only)	No primary mental-health target outcome — only nomophobia / digital literacy
Doğan Laçin BG	2025	Türkiye	Cross-sectional relational/predictive	General adults	Turkish adult convenience sampling	348 (3 univariate outliers removed)	NR (18-50)	18-50	68.1%	Doomscrolling Scale (Sharma 2022 / Turkish: Satici 2022)	Yes	—	83.6% single; 71.3% unemployed
Kaya B	2025	Türkiye	Cross-sectional with mediational SEM	General adults	Across 43 Turkish provinces (11 earthquake-affected)	415	34.98	18-66	75.4%	Doomscrolling Scale - Short Form (Sharma 2022 4-item / Turkish: Satici 2022)	Yes	—	84.8% from earthquake-affected provinces
Canoğulları Ö	2025	Türkiye	Cross-sectional SEM	University students	Turkish university students; snowball via Google Forms	460	NR (ages 18-30)	18-30	78%	Doomscrolling Scale (Turkish) (Sharma 2022 / Turkish: Satici 2023)	Yes	—	Single Turkish author from Artvin Coruh University
Hussain T	2025	Pakistan	Cross-sectional correlational	Young adults	Pakistani university students (UMT Lahore)	201	21.45	18-30	66.2%	Doomscrolling Scale (Sharma 2022)	No	Whole-study narrative-only (the available report's Table 3 did not permit reliable extraction of a	Lahore-based universities; convenience sampling

First author	Year	Country / region	Study design	Population	Recruitment setting	Sample size	Mean age	Age range	Female (%)	Doomscrolling measure	Included in primary meta	Narrative/partial status	Notes
												directly comparable zero-order correlation; reliability coefficient not reported)	

Supplementary Table S1b. Outcome-level extracted effects

Note. Each row is a study × outcome effect. Forty primary effects across anxiety, depression, psychological distress, psychological well-being, and life satisfaction enter the five prespecified primary pools. Pearson r and eligible Spearman rho estimates were synthesized on the Fisher-z scale. Adjusted, domain-specific, duplicated, sparse, or otherwise noncomparable effects were retained narratively or as nonprimary quantitative evidence. The Yang et al. sleep/insomnia association was not reverse-coded by assumption because scoring direction could not be verified. The Soraci DSS-15 psychological-distress association ($r = 0.658$, $N = 300$) is included exactly once; no DSS-4 criterion-correlation matrix suitable for the primary outcome pools was reported.

First author (Year)	Outcome domain	Outcome instrument	Effect-size type	Effect-size value	N for effect	p value	Extractable zero-order correlation	Included in primary meta-analysis	Reason not included	Direction	Notes
Ulaş E (2026)	PTSD symptoms	Post-Earthquake Trauma Scale	Pearson r	0.650	414	<0.01	Yes	No	Secondary outcome / sparse domain	Positive	Zero-order correlation
Ulaş E (2026)	Other psychological outcome	Future Time Perspective Scale - Opportunities subdim	Pearson r	-0.468	414	<0.01	No	No	Not in target outcome list (Other psychological — future time perspective)	Negative	Negative correlation with doomscrolling
Ulaş E (2026)	Other psychological outcome	Future Time Perspective Scale - Limitations subdim	Pearson r	0.573	414	<0.01	No	No	Not in target outcome list	Positive	Positive correlation with doomscrolling
Ulaş E (2026)	Problematic social media use	Problematic Social Media Use Scale	Pearson r	0.583	414	<0.01	Yes	No	Adjacent construct	Positive	Strongly correlated with doomscrolling
Camadan F (2025)	Sleep	Pittsburgh Sleep Quality Index (PSQI)	Unstandardized regression b	0.059	663	NR	No	No	Unstandardized regression coefficient; not directly comparable to a zero-order correlation.	Positive	Total effect of doomscrolling on sleep quality
Camadan F (2025)	Fear of missing out	FoMO Scale	Unstandardized regression b	0.279	663	NR	No	No	Unstandardized regression coefficient; not directly comparable to a zero-order correlation.	Positive	Doomscrolling -> FoMO path
Camadan F (2025)	Other psychological outcome	Nomophobia Scale	Unstandardized regression b	0.092	663	NR	No	No	Unstandardized regression coefficient; not directly comparable to a zero-order correlation.	Positive	Doomscrolling -> nomophobia path
Ang CS (2025)	Depression	DASS-21 Depression subscale	Pearson r	0.43	235	<0.001	Yes	Yes	—	Positive	Zero-order
Ang CS (2025)	Anxiety	DASS-21 Anxiety subscale	Pearson r	0.22	235	<0.001	Yes	Yes	—	Positive	Zero-order

First author (Year)	Outcome domain	Outcome instrument	Effect-size type	Effect-size value	N for effect	p value	Extractable zero-order correlation	Included in primary meta-analysis	Reason not included	Direction	Notes
Ang CS (2025)	Stress	DASS-21 Stress subscale	Pearson r	0.15	235	<0.05	Yes	No	Secondary outcome / sparse domain	Positive	Zero-order
Ang CS (2025)	Secondary traumatic stress	STSS-SM (Secondary Traumatic Stress Scale for Social Media Users)	Pearson r	0.16	235	<0.05	Yes	No	Secondary outcome / sparse domain	Positive	Zero-order
Yıldırım U (2025)	Life satisfaction	Satisfaction with Life Scale (Turkish)	Pearson r	-0.244	380	<0.01	Yes	Yes	—	Negative	Zero-order
Yıldırım U (2025)	Positive affect	Positivity Scale (Turkish)	Pearson r	-0.211	380	<0.01	Yes	No	Secondary outcome / sparse domain	Negative	Positivity treated as positive affect; zero-order
Dominguez-Rodriguez A (2025)	Depression	Beck Depression Inventory (BDI)	Unstandardized regression b	-0.01	292	0.148	No	No	Unstandardized regression coefficient; not directly comparable to a zero-order correlation.	Non-significant	BDI did not significantly predict doomscrolling in adjusted analysis
Dominguez-Rodriguez A (2025)	Anxiety	Hamilton Rating Scale for Anxiety (HAM-A)	Unstandardized regression b	-0.0002	292	0.988	No	No	Unstandardized regression coefficient; not directly comparable to a zero-order correlation.	Non-significant	Statement of positive association in abstract conflicts with non-sig adjusted regression
Dominguez-Rodriguez A (2025)	Other psychological outcome	Climate Change-specific Doomscrolling Scale (CCDS)	Pearson r	0.36	365	<0.001	No	No	Secondary outcome (not in primary target set); retained for narrative synthesis	Positive	Moderate correlation between DS and CCDS
Yang L (2024)	Depression	Patient Health Questionnaire (PHQ-9)	Pearson r	0.346	2885	<0.01	Yes	Yes	—	Positive	DS-15 with PHQ-9. Direction confirmed for depression; restored to primary meta-analysis.
Yang L (2024)	Anxiety	Generalized Anxiety Disorder Scale (GAD-7)	Pearson r	0.370	2885	<0.01	Yes	Yes	—	Positive	DS-15 with GAD-7. Direction confirmed for anxiety; restored to primary meta-analysis.
Yang L (2024)	Other psychological outcome	Smartphone Addiction Scale-Short	Pearson r	0.450	2885	<0.01	No	No	Not a primary target (closer to social media addiction)	Positive	DS-15 with SAS-SV

First author (Year)	Outcome domain	Outcome instrument	Effect-size type	Effect-size value	N for effect	p value	Extractable zero-order correlation	Included in primary meta-analysis	Reason not included	Direction	Notes
		(SAS-SV)									
Yang L (2024)	Sleep	Sleep Condition Indicators	Spearman rho	-0.47	578	<0.01	No	No	Scoring direction could not be verified from the report or supplement; no assumption-based recoding was undertaken.	Negative (higher SCI = less insomnia → less DS)	Retained for narrative synthesis; Spearman-based estimate not included in the primary pool.
McCutcheon LE (2024)	Depression	Depression Subscale of Brief Symptom Inventory (BSI)	Pearson r	0.29	627	<0.01	Yes	Yes	—	Positive (Iran)	IRAN subsample
McCutcheon LE (2024)	Anxiety	Dark Future Scale (DFS) - future anxiety	Pearson r	0.32	627	<0.01	Yes	Yes	—	Positive (Iran)	IRAN subsample
McCutcheon LE (2024)	Psychological well-being	Psychological Well-being Scale (PWS)	Pearson r	-0.19	627	<0.01	Yes	Yes	—	Negative (Iran)	IRAN subsample
McCutcheon LE (2024)	Depression	Depression Subscale of BSI	Pearson r	0.07	185	p>0.05 (NS)	Yes	Yes	—	Non-significant (US)	US subsample
McCutcheon LE (2024)	Anxiety	Dark Future Scale (DFS)	Pearson r	0.34	185	<0.01	Yes	Yes	—	Positive (US)	US subsample
McCutcheon LE (2024)	Psychological well-being	Psychological Well-being Scale (PWS)	Pearson r	-0.30	185	<0.01	Yes	Yes	—	Negative (US)	US subsample
McCutcheon LE (2024)	Other psychological outcome	Celebrity Attitude Scale (CAS)	Pearson r	0.20 (Iran); 0.21 (US)	627 / 185	<0.05	No	No	Not target outcome (celebrity worship)	Positive	Both samples
Taskin S (2024)	Psychological well-being	Warwick-Edinburgh Mental Well-being Scale (WEMWBS, Turkish)	Pearson r	-0.235	400	<0.01	Yes	Yes	—	Negative	Zero-order
Taskin S (2024)	Secondary traumatic stress	STSS Turkish	Pearson r	0.424	400	<0.01	Yes	No	Secondary outcome / sparse domain	Positive	Zero-order
Taskin S (2024)	Other	Mindfulness scale	Pearson r	-0.198	400	<0.01	No	No	Mindfulness not in	Negative	Zero-order

First author (Year)	Outcome domain	Outcome instrument	Effect-size type	Effect-size value	N for effect	p value	Extractable zero-order correlation	Included in primary meta-analysis	Reason not included	Direction	Notes
	psychological outcome								target outcomes (Other psychological)		
Kartol A (2025)	Psychological well-being	Well-being Scale	Standardized regression β	-0.183	508	<0.001	No	No	Standardized β only; not directly comparable to Pearson r	Negative	The available report did not permit reliable extraction of a directly comparable DS–well-being zero-order correlation; retained as nonprimary evidence.
Kartol A (2025)	Other psychological outcome	Death Distress Scale	Standardized regression β	-0.172	508	<0.001	No	No	Standardized β only; not directly comparable to Pearson r	Negative	Zero-order r with DS not reported
Kartol A (2023)	Anxiety	Dark Future Scale (Future Anxiety)	Pearson r	0.536	402	<0.01	Yes	Yes	—	Positive	Zero-order; future anxiety as anxiety subdomain
Kartol A (2023)	Psychological distress	Psychological Distress Scale	Pearson r	0.470	402	<0.01	Yes	Yes	—	Positive	Zero-order
Satıcı SA (2023)	Social media addiction	Bergen Social Media Addiction Scale (BSMAS)	Pearson r	0.358	419	<0.01	Yes	No	Adjacent construct	Positive	Study II sample
Satıcı SA (2023)	Fear of missing out	Fear of Missing Out Scale (FoMOS)	Pearson r	0.377	419	<0.01	Yes	No	Narrative/nonprimary quantitative evidence	Positive	Study II sample
Satıcı SA (2023)	Other psychological outcome	Big Five Inventory (Conscientiousness)	Pearson r	-0.168	419	<0.01	No	No	Personality trait, not eligible outcome	Negative	Study II personality correlation
Satıcı SA (2023)	Other psychological outcome	Big Five Inventory (Neuroticism)	Pearson r	0.217	419	<0.01	No	No	Personality trait	Positive	Study II
Satıcı SA (2023)	Life satisfaction	Satisfaction with Life Scale (SWLS-3)	Pearson r	-0.290	460	<0.01	Yes	Yes	—	Negative	Study III sample (separate from Study II)
Satıcı SA (2023)	Psychological well-being	Short Warwick-Edinburgh Mental Wellbeing Scale (SWEMS)	Pearson r	-0.296	460	<0.01	Yes	Yes	—	Negative	Study III
Satıcı SA (2023)	Psychological distress	DASS-21 total	Pearson r	0.391	460	<0.01	Yes	Yes	—	Positive	Study III

First author (Year)	Outcome domain	Outcome instrument	Effect-size type	Effect-size value	N for effect	p value	Extractable zero-order correlation	Included in primary meta-analysis	Reason not included	Direction	Notes
Satıcı SA (2023)	Other psychological outcome	Harmony in Life Scale (HILS-3)	Pearson r	-0.290	460	<0.01	No	No	Harmony in life not in target outcome list (related to well-being but separate construct)	Negative	Study III
Buchanan K (2021)	Positive affect	Scale of Positive and Negative Experience (SPANE-Positive)	Mean difference t-test (Cohen's d)	d=0.42 (Study 1); d=0.40 (Study 2)	Study 1 299; Study 2 603	<0.001 / 0.01	No (experimental, not correlational)	No	Experimental mean difference / Cohen's d; not pooled with correlational meta	Negative (COVID condition lower than control)	Experimental
Buchanan K (2021)	Negative affect	SPANE-Negative	Mean difference t-test/ANOVA (Cohen's d)	d=0.24 (Study 1); d=0.36 (Study 2)	Study 1 299; Study 2 603	0.28 / <0.001	No	No	Experimental mean difference / Cohen's d; not pooled with correlational meta	Positive (COVID condition higher)	Experimental
Buchanan K (2021)	Other psychological outcome	State Optimism Measure (SOM)	Mean difference t-test/ANOVA (Cohen's d)	d=0.11 (Study 1 NS); d=0.28 (Study 2)	Study 1 299; Study 2 603	0.99 / 0.001	No	No	Experimental mean difference / Cohen's d; not pooled with correlational meta	Negative (Study 2 sig)	Experimental
Antonysamy S (2026)	Psychological well-being	Flourishing Scale (FS)	Pearson r	-0.524	399	<0.001	Yes	Yes	—	Negative	Zero-order
Antonysamy S (2026)	Social media addiction	SNS Addiction Scale	Pearson r	0.456	399	<0.001	Yes	No	Adjacent construct	Positive	Zero-order
Antonysamy S (2026)	Other psychological outcome	Nomophobia Questionnaire	Pearson r	0.379	399	<0.001	No	No	Nomophobia not in primary target list (Other psychological)	Positive	Zero-order
Kaya B (2025)	Psychological well-being	Psychological Well-Being Scale (Diener; Turkish: Telef)	Pearson r	-0.22	496	<0.01	Yes	Yes	—	Negative	Zero-order
Kaya B (2025)	Other psychological outcome	Ruminative Response Scale Short Form (RRS-SF)	Pearson r	0.33	496	<0.01	Yes	No	Narrative/nonprimary quantitative evidence	Positive	Zero-order
Kaya B (2025)	Other	Intolerance of	Pearson r	0.16	496	<0.01	No	No	IUS not in target	Positive	Zero-order

First author (Year)	Outcome domain	Outcome instrument	Effect-size type	Effect-size value	N for effect	p value	Extractable zero-order correlation	Included in primary meta-analysis	Reason not included	Direction	Notes
	psychological outcome	Uncertainty Scale (IUS-12)							outcomes		
Soraci P (2025)	Stress	DASS-21 Stress	Spearman rho	0.550	300	<0.001	Yes	No	Secondary outcome / sparse domain	Positive	Zero-order Spearman-based estimate; Pearson-only sensitivity analyses were completed as specified.
Soraci P (2025)	Anxiety	DASS-21 Anxiety	Spearman rho	0.597	300	<0.001	Yes	Yes	—	Positive	Zero-order Spearman-based estimate; Pearson-only sensitivity analyses were completed as specified.
Soraci P (2025)	Depression	DASS-21 Depression	Spearman rho	0.586	300	<0.001	Yes	Yes	—	Positive	Zero-order Spearman-based estimate; Pearson-only sensitivity analyses were completed as specified.
Soraci P (2025)	Life satisfaction	Satisfaction with Life Scale (SWLS)	Spearman rho	-0.384	300	<0.001	Yes	Yes	—	Negative	Zero-order Spearman-based estimate; Pearson-only sensitivity analyses were completed as specified.
Soraci P (2025)	Psychological well-being	Warwick-Edinburgh Mental Well-being Scale (WEMWBS)	Spearman rho	-0.493	300	<0.001	Yes	Yes	—	Negative	Zero-order Spearman-based estimate; Pearson-only sensitivity analyses were completed as specified.
Soraci P (2025)	Problematic social media use	Bergen Social Media Addiction Scale (BSMAS)	Spearman rho	0.575	300	<0.001	Yes	No	Adjacent construct	Positive	Zero-order Spearman-based estimate; Pearson-only sensitivity analyses were completed as specified.
Soraci P (2025)	Psychological distress	DASS-21 total psychological distress (observed composite)	Pearson r	0.658	300	<0.001	Yes	Yes	—	Positive	DSS-15 observed criterion correlation reported in Supplementary Table S5 and included exactly once in the psychological-distress primary pool. No DSS-4 criterion-correlation matrix suitable for the primary outcome pools was reported.
Blacha S (2024)	Life satisfaction	Satisfaction with Life Scale (SWLS)	Pearson r	-0.12	677	<0.01	Yes	Yes	—	Negative	Zero-order

First author (Year)	Outcome domain	Outcome instrument	Effect-size type	Effect-size value	N for effect	p value	Extractable zero-order correlation	Included in primary meta-analysis	Reason not included	Direction	Notes
Blacha S (2024)	Other psychological outcome	Social Connectedness Scale (van Bel 2009)	Pearson r	-0.15	677	<0.01	No	No	Social connectedness not in target	Negative	Zero-order
Blacha S (2024)	Other psychological outcome	Brief Hypervigilance Scale (Bernstein 2015)	Pearson r	0.42	677	<0.001	No	No	Hypervigilance not target	Positive	Zero-order
Kaya B (2025)	Psychological well-being	Warwick-Edinburgh Mental Wellbeing Scale Short Form (WEMWBS-SF)	Pearson r	-0.296	432	<0.01	Yes	Yes	—	Negative	Zero-order
Kaya B (2025)	Other psychological outcome	Intolerance of Uncertainty Scale (IUS-12)	Pearson r	0.329	432	<0.01	No	No	Not target	Positive	Zero-order
Kurtça TT (2025)	Anxiety	Generalized Anxiety Disorder Scale (GAD-7)	Pearson r	0.34	553	<0.001	Yes	Yes	—	Positive	Zero-order
Kurtça TT (2025)	Social media addiction	Social Media Addiction Scale	Pearson r	0.30	553	<0.001	Yes	No	Adjacent construct	Positive	Zero-order
Kurtça TT (2025)	Other psychological outcome	Brief Resilience Scale (Psychological Resilience)	Pearson r	-0.29	553	<0.001	No	No	Resilience not in primary target list	Negative	Zero-order
Kurtça TT (2025)	Other psychological outcome	Cyberchondria Severity	Pearson r	0.37	553	<0.001	No	No	Cyberchondria not target	Positive	Zero-order
Shabahang R (2023)	Depression	Brief Symptom Inventory (BSI) Depression	Pearson r	0.32	707	<0.001	Yes	Yes	—	Positive	Zero-order
Shabahang R (2023)	Psychological well-being	Psychological Well-Being Scale (8-item)	Pearson r	-0.30	707	<0.001	Yes	Yes	—	Negative	Zero-order
Shabahang R (2023)	Life satisfaction	Satisfaction with Life Scale (SWLS)	Pearson r	-0.24	707	<0.001	Yes	Yes	—	Negative	Zero-order
Shabahang R (2023)	Anxiety	Dark Future Scale	Pearson r	0.33	707	<0.001	Yes	Yes	—	Positive	Zero-order

First author (Year)	Outcome domain	Outcome instrument	Effect-size type	Effect-size value	N for effect	p value	Extractable zero-order correlation	Included in primary meta-analysis	Reason not included	Direction	Notes
		(DFS) - future anxiety									
Körün AB (2025)	Psychological well-being	Mental Well-Being Scale (Turkish)	Standardized regression β	-0.36	811	<0.01	No	No	Standardized β only; not directly comparable to Pearson r	Negative	Published Table 2 was available, but the report did not permit reliable extraction of a directly comparable zero-order correlation; retained as nonprimary evidence.
Körün AB (2025)	Other psychological outcome	Family Functioning Scale	Standardized regression β	-0.15	811	<0.01	No	No	Standardized β only; not directly comparable to Pearson r	Negative	SEM only
Körün AB (2025)	Other psychological outcome	Schadenfreude Scale	Standardized regression β	0.72	811	<0.01	No	No	Standardized β only; not directly comparable to Pearson r	Positive	SEM only
Shabahang R (2024)	Anxiety	Existential Anxiety Questionnaire (EAQ) — Iran subsample	Pearson r	0.28	620	<0.01	Yes	Yes	—	Positive	Zero-order
Shabahang R (2024)	Anxiety	Existential Anxiety Questionnaire (EAQ) — US subsample	Pearson r	0.30	180	<0.01	Yes	Yes	—	Positive	Zero-order
Shabahang R (2024)	Other psychological outcome	Misanthropy Scale — Iran	Pearson r	0.21	620	<0.01	No	No	Misanthropy not target outcome	Positive	Zero-order
Shabahang R (2024)	Other psychological outcome	Misanthropy Scale — US	Pearson r	0.30	180	<0.01	No	No	Misanthropy not target	Positive	Zero-order
Shabahang R (2024)	Other psychological outcome	Global Belief in Just World Scale (GBJWS) — Iran	Pearson r	-0.10	620	p>0.01 (NS)	No	No	Just-world belief not target; non-sig	Non-significant	Zero-order
Shabahang R (2024)	Other psychological outcome	Global Belief in Just World Scale (GBJWS) — US	Pearson r	0.10	180	p>0.01 (NS)	No	No	Just-world belief not target; non-sig	Non-significant	Zero-order
Kütük H (2025)	Psychological	Brief Psychological	Pearson r	-0.335	406	<0.001	Yes	Yes	—	Negative	Zero-order

First author (Year)	Outcome domain	Outcome instrument	Effect-size type	Effect-size value	N for effect	p value	Extractable zero-order correlation	Included in primary meta-analysis	Reason not included	Direction	Notes
	well-being	Adjustment Scale (BPAS, Cruz 2020)									
Kütük H (2025)	Other psychological outcome	Beck Hopelessness Scale (Turkish)	Pearson r	0.319	406	<0.001	Yes	No	Narrative/nonprimary quantitative evidence	Positive	Zero-order
Kütük H (2025)	Other psychological outcome	Intolerance of Uncertainty Scale (IUS-12)	Pearson r	0.315	406	<0.001	No	No	IUS not target outcome	Positive	Zero-order
Sharma B (2022)	Anxiety	Generalized Anxiety Disorder-7 (GAD-7)	Pearson r	0.307	502	<0.001	Yes	Yes	—	Positive	Zero-order
Sharma B (2022)	Psychological well-being	World Health Organization-5 Well-Being Index (WHO-5)	Pearson r	-0.005	502	0.909 (NS)	Yes	Yes	—	Non-significant	Zero-order; statistically non-significant
Sharma B (2022)	Fear of missing out	FoMO Scale (Przybylski 2013)	Pearson r	0.438	502	<0.001	Yes	No	Narrative/nonprimary quantitative evidence	Positive	Zero-order
Sharma B (2022)	Problematic social media use	Bergen Social Media Addiction Scale (BSMAS)	Pearson r	0.550	502	<0.001	Yes	No	Adjacent construct	Positive	Zero-order
Sharma B (2022)	Positive affect	Scale of Positive and Negative Experience (SPANE - combined)	Pearson r	-0.185	502	<0.001	Yes	No	Secondary outcome / sparse domain	Negative	Zero-order
Sharma B (2022)	Problematic social media use	Generalized Problematic Internet Use Scale (GPIUS2)	Pearson r	0.482	502	<0.001	Yes	No	Adjacent construct	Positive	Zero-order
Hughes IM (2024)	Other psychological outcome	Work-Related Rumination (Matthews single item)	Unstandardized regression b	0.39	352	<0.01	No	No	Unstandardized regression coefficient; not directly comparable to a zero-order correlation.	Positive	Rumination in target outcomes; but b coefficient not r
Hughes IM (2024)	Other	Work Engagement	Unstandardized	-0.13	352	<0.05	No	No	Unstandardized	Negative	Work engagement; indirect effect

First author (Year)	Outcome domain	Outcome instrument	Effect-size type	Effect-size value	N for effect	p value	Extractable zero-order correlation	Included in primary meta-analysis	Reason not included	Direction	Notes
	psychological outcome	(UWES-3)	regression b (indirect)						regression coefficient; not directly comparable to a zero-order correlation.	(indirect via rumination)	
Hughes IM (2024)	Other psychological outcome	Daily Work-Related Rumination	Multilevel γ	0.77	126 individuals / 630 obs	<0.01	No	No	Multilevel γ coefficient; not directly comparable to between-person correlations	Positive (within-person)	Within-person daily effect
Hughes IM (2024)	Other psychological outcome	Daily Work Engagement (UWES-3)	Multilevel γ	-0.16	126 / 630	<0.05	No	No	Multilevel γ coefficient; not directly comparable to between-person correlations	Negative	Within-person
Türk-Kurtça T (2025)	Anxiety	Trait Anxiety Scale (STAI-T, Spielberger 1983)	Pearson r	0.214	443	<0.001	Yes	Yes	—	Positive	Zero-order
Türk-Kurtça T (2025)	Other psychological outcome	Brief Resilience Scale (BRS, Smith 2008)	Pearson r	-0.265	443	<0.001	No	No	Resilience not target	Negative	Zero-order
Türk-Kurtça T (2025)	Other psychological outcome	Intolerance of Uncertainty Scale (IUS-12)	Pearson r	0.310	443	<0.001	No	No	IUS not target	Positive	Zero-order
Biricik ZD (2026)	Other psychological outcome	Nomophobia Questionnaire (NMP-Q) overall	Pearson r	0.380	779	<0.001	No	No	Nomophobia not in primary target outcomes; only relevant if expanding scope	Positive	Zero-order; no other target outcomes measured
Biricik ZD (2026)	Other psychological outcome	Digital Literacy Scale	Pearson r	0.001	779	0.977 (NS)	No	No	Not target outcome	Non-significant	Null finding
Doğan Laçin BG (2025)	Psychological well-being	Psychological Well-Being Scale (Diener 2010; Turkish: Telef 2013)	Pearson r	-0.263	348	<0.005	Yes	Yes	—	Negative	Zero-order

First author (Year)	Outcome domain	Outcome instrument	Effect-size type	Effect-size value	N for effect	p value	Extractable zero-order correlation	Included in primary meta-analysis	Reason not included	Direction	Notes
Doğan Laçin BG (2025)	Emotion regulation	Emotion Regulation Scale - Suppression subscale (John & Gross 2003)	Pearson r	0.207	348	<0.005	Yes	No	Secondary outcome / sparse domain	Positive	Zero-order
Doğan Laçin BG (2025)	Emotion regulation	Emotion Regulation Scale - Reappraisal subscale	Pearson r	-0.096	348	<0.005	Yes	No	Secondary outcome / sparse domain	Weak negative	Zero-order
Kaya B (2025)	Psychological distress	DASS-21 total (Henry & Crawford 2005)	Pearson r	0.50	415	<0.01	Yes	Yes	—	Positive	Zero-order
Kaya B (2025)	Other psychological outcome	Fear of Earthquake Scale (adapted from FCV-19S)	Pearson r	0.37	415	<0.01	No	No	Fear of earthquake not in target outcomes (it's a stressor, not the outcome of doomscrolling)	Positive	Zero-order
Kaya B (2025)	Other psychological outcome	Intolerance of Uncertainty Scale (IUS-12)	Pearson r	0.32	415	<0.01	No	No	IUS not target	Positive	Zero-order
Canoğulları Ö (2025)	Psychological distress	DASS-21 total	Pearson r	0.49	460	<0.01	Yes	Yes	—	Positive	Zero-order
Canoğulları Ö (2025)	Social media addiction	Bergen Social Media Addiction Scale (BSMAS)	Pearson r	0.34	460	<0.01	Yes	No	Adjacent construct	Positive	Zero-order
Hussain T (2025)	Burnout	Digital Burnout Scale (Erten & Ozdemir 2020)	Pearson r	0.41	201	<0.01	No	No	Secondary outcome (not in primary target set); retained for narrative synthesis	Positive	The available report did not permit reliable extraction of a directly comparable zero-order correlation; retained as nonprimary evidence.
Hussain T (2025)	Other psychological outcome	Multiple Screen Addiction Scale (Saritepeci 2021) — overall	Pearson r	0.23	201	<0.01	No	No	Secondary outcome (not in primary target set); retained for narrative synthesis	Positive	Zero-order
Hussain T (2025)	Burnout	Digital Burnout - Digital Aging subscale	Pearson r	0.34	201	<0.01	No	No	Secondary outcome (not in primary target set); retained for narrative synthesis	Positive	Zero-order; subscale

First author (Year)	Outcome domain	Outcome instrument	Effect-size type	Effect-size value	N for effect	p value	Extractable zero-order correlation	Included in primary meta-analysis	Reason not included	Direction	Notes
Hussain T (2025)	Burnout	Digital Burnout - Emotional Exhaustion subscale	Pearson r	0.81	201	<0.01	No	No	Secondary outcome (not in primary target set); retained for narrative synthesis	Positive	The reported subscale coefficient was retained as nonprimary evidence and did not enter a primary pool.

Supplementary Table S1c. Fourteen additional reports retained narratively

Note. These reports complete the 44-report evidence base. They were critically appraised and retained for narrative or nonprimary interpretation but did not contribute a qualifying primary effect to the five pooled outcome domains.

Report ID	Author/year	Study design	Sample size	Evidence role	Critical-appraisal tool	Full citation
FINAL_SYSREV_001	Lissitsa S 2026	Analytical cross-sectional survey	570	Narrative/nonprimary; no qualifying primary pooled effect	JBI Critical Appraisal Checklist for Analytical Cross-Sectional Studies (legacy 8-item, JBI 2020)	Sabina Lissitsa; Maya Kagan (2026). Adverse childhood experiences, loneliness, and doomscrolling on social media newsfeeds among adult men across Generations X, Y, Z. Frontiers in Sociology. 11:1700210. https://doi.org/10.3389/fsoc.2026.1700210.
FINAL_SYSREV_002	Dyar C 2024	Prospective 30-day ecological momentary assessment cohort	429	Narrative/nonprimary; no qualifying primary pooled effect	JBI Critical Appraisal Checklist for Cohort Studies (11-item, JBI 2020)	Christina Dyar; Shariell Crosby; Michael E. Newcomb; Brian Mustanski; Debra Kaysen (2024). Doomscrolling: Prospective associations between daily COVID news exposure, internalizing symptoms, and substance use among sexual and gender minority individuals assigned female at birth. Psychology of Sexual Orientation and

Report ID	Author/year	Study design	Sample size	Evidence role	Critical-appraisal tool	Full citation
						Gender Diversity. 11(1):139-152. https://doi.org/10.1037/sgd0000585.
FINAL_SYSREV_003	[Article in Russian 2023	Analytical cross-sectional population survey	7777	Narrative/nonprimary; no qualifying primary pooled effect	JBI Critical Appraisal Checklist for Analytical Cross-Sectional Studies (legacy 8-item, JBI 2020)	T.S. Syunyakov; A.V. Zakharov; A.J. Gayduk; J.S. Ignatenko; N.Y. Kuvshinova; A.V. Pavlichenko; A.A. Spikina; I.A. Fedotov; A.A. Yashikhina; X. Gonda; A. Desousa; K.N. Fountoulakis; D.A. Smirnova (2023). Changes in sleep patterns and the doom-scrolling (doom-surfing) phenomenon as modifiable risk factors for anxiety due to continuous stress of the COVID-19 pandemic. S.S. Korsakov Journal of Neurology and Psychiatry. 123(10):88. https://doi.org/10.17116/jnevro202312310188.
FINAL_SYSREV_004	Conroy RM 2023	Analytical cross-sectional scale-development survey	649	Narrative/nonprimary; no qualifying primary pooled effect	JBI Critical Appraisal Checklist for Analytical Cross-Sectional Studies (legacy 8-item, JBI 2020)	Ronán M. Conroy; Karen Fitzgerald (2023). The lockdown experience scale for students (LESS). BMC Medical Education. 23(1):829. https://doi.org/10.1186/s12909-022-03858-x.
FINAL_SYSREV_005	Price M 2022	Prospective 30-day daily-diary cohort	61 analyzed (74 initial)	Narrative/nonprimary; no qualifying primary pooled	JBI Critical Appraisal Checklist for Cohort Studies	Matthew Price; Alison C. Legrand; Zoe M. F. Brier;

Report ID	Author/year	Study design	Sample size	Evidence role	Critical-appraisal tool	Full citation
				effect	(11-item, JBI 2020)	Katherine van Stolk-Cooke; Kelly Peck; Peter Sheridan Dodds; Christopher M. Danforth; Zachary W. Adams (2022). Doomscrolling during COVID-19: The negative association between daily social and traditional media consumption and mental health symptoms during the COVID-19 pandemic. Psychological Trauma: Theory, Research, Practice, and Policy. 14(8):1338-1346. https://doi.org/10.1037/tra0001202.
FINAL_SYSREV_006	Flack 2024	Analytical cross-sectional mediation survey	603	Narrative/nonprimary; no qualifying primary pooled effect	JBI Critical Appraisal Checklist for Analytical Cross-Sectional Studies (legacy 8-item, JBI 2020)	Mal Flack; William H Burton; Kim M Caudwell (2024). I rely on a little help from my friends: the effect of interpersonal and intrapersonal emotion regulation on the relationship between FOMO and problematic internet use. BMC Psychiatry. 24(1):384. https://doi.org/10.1186/s12888-024-05834-9.
FINAL_SYSREV_007	Maksimenko 2024	Analytical cross-sectional validation/correlational survey	1025	Narrative/nonprimary; no qualifying primary pooled effect	JBI Critical Appraisal Checklist for Analytical Cross-Sectional Studies (legacy 8-item, JBI 2020)	A. A. Maksimenko; A. A. Zolotareva (2024). The online literacy of Russian citizen in issues of health

Report ID	Author/year	Study design	Sample size	Evidence role	Critical-appraisal tool	Full citation
						information: psychometric characteristics of Russian version of eHEALS. Problems of Social Hygiene Public Health and History of Medicine. 32(6). https://doi.org/10.32687/0869-866x-2024-32-6-1314-1320.
FINAL_SYSREV_008	Kiye 2024	Analytical cross-sectional latent-profile survey	572 analyzed	Narrative/nonprimary; no qualifying primary pooled effect	JBI Critical Appraisal Checklist for Analytical Cross-Sectional Studies (legacy 8-item, JBI 2020)	Semra Kiye; Betul Gokcen Dogan; Fatma Coşkun (2024). Latent profile analysis of earthquake victims: doomscrolling, psychological well-being, emotion regulation and resilience. Current Psychology. 43(44):33867-33877. https://doi.org/10.1007/s12144-024-06904-7.
FINAL_SYSREV_009	Rajan 2026	Analytical cross-sectional survey	331	Narrative/nonprimary; no qualifying primary pooled effect	JBI Critical Appraisal Checklist for Analytical Cross-Sectional Studies (legacy 8-item, JBI 2020)	C S Karthik Rajan; P. Keerthana; Sujatha Sathiya (2026). Personality and doomscrolling: The mediating role of fear of missing out in an Eastern context. New Ideas in Psychology. 80:101208. https://doi.org/10.1016/j.newideapsych.2025.101208.
FINAL_SYSREV_010	Kaye 2024	Analytical cross-sectional national panel survey	684	Narrative/nonprimary; no qualifying primary pooled effect	JBI Critical Appraisal Checklist for Analytical Cross-Sectional Studies	Barbara K. Kaye; Thomas J. Johnson (2024). I can't stop myself! Doomscrolling,

Report ID	Author/year	Study design	Sample size	Evidence role	Critical-appraisal tool	Full citation
					(legacy 8-item, JBI 2020)	conspiracy theories, and trust in social media. Atlantic Journal of Communication. 32(3):471-483. https://doi.org/10.1080/15456870.2024.2316844.
FINAL_SYSREV_011	P Singh 2024	Analytical cross-sectional correlational survey	30	Narrative/nonprimary; no qualifying primary pooled effect	JBI Critical Appraisal Checklist for Analytical Cross-Sectional Studies (legacy 8-item, JBI 2020)	Prabhsimran Singh; Aashna Narula (2024). A Study on Doom Scrolling Behavior and Its Correlation with Personality types and Psychological Distress GenZ College Students. International Journal of Interdisciplinary Approaches in Psychology. 2(12). https://doi.org/10.61113/ijiap.v2i12.630.
FINAL_SYSREV_012	K Simon 2025	Analytical cross-sectional thesis survey; accessible PDF is incomplete	191	Narrative/nonprimary; no qualifying primary pooled effect	JBI Critical Appraisal Checklist for Analytical Cross-Sectional Studies (legacy 8-item, JBI 2020)	K. Simon (2025). Doomscrolling : A Correlational Study Testing Mood Management Theory in the Context of Mental and Social Well - Being. Master's thesis, Florida State University, College of Communication & Information.
FINAL_SYSREV_013	MRB Manon-og 2026	Analytical cross-sectional descriptive-correlational survey	51	Narrative/nonprimary; no qualifying primary pooled effect	JBI Critical Appraisal Checklist for Analytical Cross-Sectional Studies (legacy 8-item, JBI 2020)	Maiah Reva B. Manon-og; Gogen P. Antipuesto; Jhun Bryan D. Mehoy; Edeva Joy Dybongco; Gretel T. Ricalde (2026). DOOMSCROLLING

Report ID	Author/year	Study design	Sample size	Evidence role	Critical-appraisal tool	Full citation
						AND DEPRESSIVE SYMPTOMS: ITS IMPACT ON MENTAL HEALTH. Ignatian International Journal for Multidisciplinary Research. 4(2):1233-1246. https://doi.org/10.5281/zenodo.18814937.
FINAL_SYSREV_014	KTAN YILDIRIM 2026	Analytical cross-sectional mediation survey (preprint)	400	Narrative/nonprimary; no qualifying primary pooled effect	JBI Critical Appraisal Checklist for Analytical Cross-Sectional Studies (legacy 8-item, JBI 2020)	Kübra TAN YILDIRIM; Vesile ADIGÜZEL; Yalçın KANBAY (2026). The Mediating Role of Doomscrolling in the Relationship Between Perceptual– Concentration Errors and Anxiety. Research Square preprint. https://doi.org/10.21203/rs.3.rs-9372297/v1.

Supplementary Table S1d. Field definitions

Term	Definition
Source stream	Database/register searches plus supplementary Google Scholar screening of the first 200 relevance-ranked records, with deduplication and citation searching as reported in Supplementary Methods 1 and Figure 1.
Study design	Cross-sectional (most), cross-sectional with mediation/SEM, multi-study cross-sectional, or experimental (Buchanan 2021 only). Sub-studies within a paper (e.g., Studies II/III of Satıcı 2023) are extracted as separate effect rows in S1b.
Doomscrolling measure	The named exposure scale and version (e.g., 'Doomscrolling Scale (Sharma 2022 / Turkish: Satıcı 2023, 15-item)'). The same instrument family appears across most studies.
Included in primary meta-analysis (S1a)	Yes = at least one outcome effect from this study enters the primary meta-analysis pool. No = all outcome effects are narrative-only. Partial = some outcomes are pooled and some are retained narratively (current pool: Yang 2024 is the only Partial study).
Effect-size type	Pearson r and Spearman rho effects enter the primary meta-analysis pool. Standardized β, unstandardized b, multilevel γ, Cohen's d, and mediation path coefficients are NOT directly poolable with

Term	Definition
(S1b)	correlational meta-analysis.
Extractable for meta-analysis (S1b)	Yes if a directly poolable zero-order r or rho is available; No otherwise. The 'Reason not included' column gives the specific reason when No.
Yang 2024 partial inclusion	Yang et al. 2024 contributes PHQ-9 depression (r=0.346) and GAD-7 anxiety (r=0.370) at N=2885 to the primary meta. The Sleep Condition Indicators correlation (ρ=−0.47 at N=578) was retained for narrative synthesis because scoring direction could not be verified from the report or supplement; no assumption-based recoding was undertaken. The Spearman-based estimate was not included in the primary pool.
Spearman rho note	Soraci et al. (2025) reported Spearman associations for the main-article anxiety, depression, life-satisfaction, psychological well-being, and stress measures. The observed DSS-15 psychological-distress correlation of 0.658 in Supplementary Table S5 is Pearson r. Eligible associations were Fisher-z transformed; Pearson-only sensitivity analyses were completed. No DSS-4 criterion-correlation matrix suitable for the primary outcome pools was reported.
Narrative-only studies	The final review includes 44 reports. Twenty-one studies contributed 40 primary effects; 23 reports supplied narrative or nonprimary quantitative evidence. The 14 reports outside the 30-report structured quantitative-extraction subset are listed in Supplementary Table S1c.

Supplementary Table S2. Complete design-appropriate JBI critical appraisal

All 46 appraisal units representing 44 included reports were assessed with design-appropriate JBI tools. Item-level judgments remain primary. Overall Low/Moderate/High categories are review-derived summaries based on the proportion of affirmative applicable items: Low ≥87.5%; Moderate ≥62.5% and <87.5%; High <62.5%. No Not-applicable judgments occurred.

Supplementary Table S2a. Appraisal-unit summary

Appraisal unit	Report(s)	Author/year	Design	JBI checklist	Yes/Total	% Yes	Overall RoB	Primary meta
AU-001	REPORT-001	Ulaş E 2026	Cross-sectional (post-earthquake)	Analytical Cross-Sectional Studies (legacy 8-item, JBI 2020)	6/8	75.0%	Moderate	No
AU-002	REPORT-002	Camadan F 2025	Cross-sectional descriptive-correlational (narrative-only — only path b reported, no zero-order r)	Analytical Cross-Sectional Studies (legacy 8-item, JBI 2020)	6/8	75.0%	Moderate	No
AU-003	REPORT-003	Ang CS 2025	Cross-sectional	Analytical Cross-Sectional Studies (legacy 8-item, JBI 2020)	6/8	75.0%	Moderate	Yes
AU-004	REPORT-004	Yıldırım U 2025	Cross-sectional (occupational — deck cadets)	Analytical Cross-Sectional Studies (legacy 8-item, JBI 2020)	6/8	75.0%	Moderate	Yes
AU-005	REPORT-005	Dominguez-Rodriguez A 2025	Cross-sectional (international online, narrative-only)	Analytical Cross-Sectional Studies (legacy 8-item, JBI 2020)	7/8	87.5%	Low	No
AU-006	REPORT-006	Yang L 2024	Cross-sectional 2-sample validation (Study 1) + mediation (Study 2). Outcome-level partial inclusion.	Analytical Cross-Sectional Studies (legacy 8-item, JBI 2020)	6/8	75.0%	Moderate	Yes
AU-007	REPORT-007	McCutcheon LE 2024	Cross-sectional cross-cultural (Iran + US)	Analytical Cross-Sectional Studies (legacy 8-item, JBI 2020)	6/8	75.0%	Moderate	Yes
AU-008	REPORT-008	Taskin S 2024	Cross-sectional with serial mediation	Analytical Cross-Sectional Studies (legacy 8-item, JBI 2020)	6/8	75.0%	Moderate	Yes
AU-009	REPORT-009	Kartol A 2025	Cross-sectional 2-phase psychometric validation (narrative-only)	Analytical Cross-Sectional Studies (legacy 8-item, JBI 2020)	5/8	62.5%	Moderate	No
AU-010	REPORT-010	Kartol A 2023	Cross-sectional predictive-correlational (post-earthquake)	Analytical Cross-Sectional Studies (legacy 8-item, JBI 2020)	8/8	100.0%	Low	Yes

Appraisal unit	Report(s)	Author/year	Design	JBI checklist	Yes/Total	% Yes	Overall RoB	Primary meta
AU-011	REPORT-011	Satıcı SA 2023	Multi-study cross-sectional scale validation (Study I N=378 qual+psychometric; Study II N=419 + Study III N=460 quantitative)	Analytical Cross-Sectional Studies (legacy 8-item, JBI 2020)	6/8	75.0%	Moderate	Yes
AU-012_STUDY1	REPORT-012	Buchanan K 2021 (Study 1)	Randomized parallel-group online experiment (Twitter feed)	Randomized Controlled Trials (13-item, JBI 2020)	5/13	38.5%	High	No
AU-012_STUDY2	REPORT-012	Buchanan K 2021 (Study 2)	Randomized parallel-group online experiment (YouTube reaction video)	Randomized Controlled Trials (13-item, JBI 2020)	5/13	38.5%	High	No
AU-013	REPORT-013	Antonysamy S 2026	Cross-sectional chain mediation	Analytical Cross-Sectional Studies (legacy 8-item, JBI 2020)	5/8	62.5%	Moderate	Yes
AU-014	REPORT-014	Kaya B (Bilal) 2025	Cross-sectional serial mediation	Analytical Cross-Sectional Studies (legacy 8-item, JBI 2020)	6/8	75.0%	Moderate	Yes
AU-015	REPORT-015	Soraci P 2025	Cross-sectional psychometric validation (Italian DSS)	Analytical Cross-Sectional Studies (legacy 8-item, JBI 2020)	5/8	62.5%	Moderate	Yes
AU-016	REPORT-016	Blacha S 2024	Cross-sectional preregistered	Analytical Cross-Sectional Studies (legacy 8-item, JBI 2020)	8/8	100.0%	Low	Yes
AU-017	REPORT-017	Kaya B (Bilal) 2025	Cross-sectional mediation + moderation	Analytical Cross-Sectional Studies (legacy 8-item, JBI 2020)	6/8	75.0%	Moderate	Yes
AU-018	REPORT-018	Kurtça TT 2025	Cross-sectional path analysis	Analytical Cross-Sectional Studies (legacy 8-item, JBI 2020)	5/8	62.5%	Moderate	Yes
AU-019	REPORT-019	Shabahang R 2023	Cross-sectional scale development + correlations	Analytical Cross-Sectional Studies (legacy 8-item, JBI 2020)	7/8	87.5%	Low	Yes
AU-020	REPORT-020	Körün AB 2025	Cross-sectional SEM + network analysis (narrative-only — PDF text-extraction corrupted)	Analytical Cross-Sectional Studies (legacy 8-item, JBI 2020)	5/8	62.5%	Moderate	No
AU-021	REPORT-021	Shabahang R 2024	Cross-sectional cross-cultural SEM	Analytical Cross-Sectional Studies (legacy 8-item, JBI 2020)	8/8	100.0%	Low	Yes
AU-022	REPORT-022	Kütük H 2025	Cross-sectional SEM	Analytical Cross-Sectional Studies	6/8	75.0%	Moderate	Yes

Appraisal unit	Report(s)	Author/year	Design	JBI checklist	Yes/Total	% Yes	Overall RoB	Primary meta
				(legacy 8-item, JBI 2020)				
AU-023	REPORT-023	Sharma B 2022	Mixed-methods multi-study (Study 1 qualitative excluded; Study 2 N=401; Study 3 N=502 — scale validation)	Analytical Cross-Sectional Studies (legacy 8-item, JBI 2020)	7/8	87.5%	Low	Yes
AU-024_STUDY1	REPORT-024	Hughes IM 2024 (Study 1)	Prospective two-timepoint time-lagged employee cohort	Cohort Studies (11-item, JBI 2020)	5/11	45.5%	High	No
AU-024_STUDY2	REPORT-024	Hughes IM 2024 (Study 2)	Prospective five-day daily-diary employee cohort	Cohort Studies (11-item, JBI 2020)	6/11	54.5%	High	No
AU-025	REPORT-025	Türk-Kurtça T 2025	Cross-sectional SEM	Analytical Cross-Sectional Studies (legacy 8-item, JBI 2020)	5/8	62.5%	Moderate	Yes
AU-026	REPORT-026	Biricik ZD 2026	Cross-sectional survey (7 Turkish regions)	Analytical Cross-Sectional Studies (legacy 8-item, JBI 2020)	6/8	75.0%	Moderate	No
AU-027	REPORT-027	Doğan Laçin BG 2025	Cross-sectional relational/predictive	Analytical Cross-Sectional Studies (legacy 8-item, JBI 2020)	7/8	87.5%	Low	Yes
AU-028	REPORT-028	Kaya B (Bilal) 2025	Cross-sectional SEM (post-earthquake)	Analytical Cross-Sectional Studies (legacy 8-item, JBI 2020)	6/8	75.0%	Moderate	Yes
AU-029	REPORT-029	Canoğulları Ö 2025	Cross-sectional SEM	Analytical Cross-Sectional Studies (legacy 8-item, JBI 2020)	8/8	100.0%	Low	Yes
AU-030	REPORT-030	Hussain T 2025	Cross-sectional correlational (narrative-only — Table 3 layout uncertainty)	Analytical Cross-Sectional Studies (legacy 8-item, JBI 2020)	5/8	62.5%	Moderate	No
AU_FINAL_SYSREV_001	FINAL_SYSREV_001	Lissitsa S 2026	Analytical cross-sectional survey	Analytical Cross-Sectional Studies (legacy 8-item, JBI 2020)	6/8	75.0%	Moderate	No
AU_FINAL_SYSREV_002	FINAL_SYSREV_002	Dyar C 2024	Prospective 30-day ecological momentary assessment cohort	Cohort Studies (11-item, JBI 2020)	8/11	72.7%	Moderate	No
AU_FINAL_SYSREV_003	FINAL_SYSREV_003	[Article in Russian 2023	Analytical cross-sectional population survey	Analytical Cross-Sectional Studies (legacy 8-item, JBI 2020)	7/8	87.5%	Low	No
AU_FINAL_SYSREV_004	FINAL_SYSREV_004	Conroy RM 2023	Analytical cross-sectional scale-development survey	Analytical Cross-Sectional Studies	5/8	62.5%	Moderate	No

Appraisal unit	Report(s)	Author/year	Design	JBI checklist	Yes/Total	% Yes	Overall RoB	Primary meta
				(legacy 8-item, JBI 2020)				
AU_FINAL_SYSREV_005	FINAL_SYSREV_005	Price M 2022	Prospective 30-day daily-diary cohort	Cohort Studies (11-item, JBI 2020)	7/11	63.6%	Moderate	No
AU_FINAL_SYSREV_006	FINAL_SYSREV_006	Flack 2024	Analytical cross-sectional mediation survey	Analytical Cross-Sectional Studies (legacy 8-item, JBI 2020)	6/8	75.0%	Moderate	No
AU_FINAL_SYSREV_007	FINAL_SYSREV_007	Maksimenko 2024	Analytical cross-sectional validation/correlational survey	Analytical Cross-Sectional Studies (legacy 8-item, JBI 2020)	7/8	87.5%	Low	No
AU_FINAL_SYSREV_008	FINAL_SYSREV_008	Kiye 2024	Analytical cross-sectional latent-profile survey	Analytical Cross-Sectional Studies (legacy 8-item, JBI 2020)	6/8	75.0%	Moderate	No
AU_FINAL_SYSREV_009	FINAL_SYSREV_009	Rajan 2026	Analytical cross-sectional survey	Analytical Cross-Sectional Studies (legacy 8-item, JBI 2020)	8/8	100.0%	Low	No
AU_FINAL_SYSREV_010	FINAL_SYSREV_010	Kaye 2024	Analytical cross-sectional national panel survey	Analytical Cross-Sectional Studies (legacy 8-item, JBI 2020)	7/8	87.5%	Low	No
AU_FINAL_SYSREV_011	FINAL_SYSREV_011	P Singh 2024	Analytical cross-sectional correlational survey	Analytical Cross-Sectional Studies (legacy 8-item, JBI 2020)	6/8	75.0%	Moderate	No
AU_FINAL_SYSREV_012	FINAL_SYSREV_012	K Simon 2025	Analytical cross-sectional thesis survey; accessible PDF is incomplete	Analytical Cross-Sectional Studies (legacy 8-item, JBI 2020)	4/8	50.0%	High	No
AU_FINAL_SYSREV_013	FINAL_SYSREV_013	MRB Manon-og 2026	Analytical cross-sectional descriptive-correlational survey	Analytical Cross-Sectional Studies (legacy 8-item, JBI 2020)	3/8	37.5%	High	No
AU_FINAL_SYSREV_014	FINAL_SYSREV_014	KTAN YILDIRIM 2026	Analytical cross-sectional mediation survey (preprint)	Analytical Cross-Sectional Studies (legacy 8-item, JBI 2020)	6/8	75.0%	Moderate	No

Supplementary Table S2b. Item-level JBI judgments

Q1–Q13 refer to the numbered items of the design-specific JBI checklist named in Supplementary Table S2a. A dash indicates that the checklist contains fewer than 13 items; it is not a Not-applicable judgment.

Appraisal unit	Q1	Q2	Q3	Q4	Q5	Q6	Q7	Q8	Q9	Q10	Q11	Q12	Q13	Yes	No	Unclear	Overall
AU-001	Yes	Yes	Yes	Yes	Unclear	Unclear	Yes	Yes	—	—	—	—	—	6	0	2	Moderate

Appraisal unit	Q1	Q2	Q3	Q4	Q5	Q6	Q7	Q8	Q9	Q10	Q11	Q12	Q13	Yes	No	Unclear	Overall
AU-002	Yes	Yes	Yes	Yes	Unclear	Unclear	Yes	Yes	—	—	—	—	—	6	0	2	Moderate
AU-003	Yes	Yes	Yes	Yes	Unclear	Unclear	Yes	Yes	—	—	—	—	—	6	0	2	Moderate
AU-004	Yes	Yes	Yes	Yes	Unclear	Unclear	Yes	Yes	—	—	—	—	—	6	0	2	Moderate
AU-005	Yes	Yes	Yes	Unclear	Yes	Yes	Yes	Yes	—	—	—	—	—	7	0	1	Low
AU-006	Yes	Yes	Yes	Yes	Unclear	Unclear	Yes	Yes	—	—	—	—	—	6	0	2	Moderate
AU-007	Yes	Yes	Yes	Yes	Unclear	Unclear	Yes	Yes	—	—	—	—	—	6	0	2	Moderate
AU-008	Yes	Yes	Yes	Yes	Unclear	Unclear	Yes	Yes	—	—	—	—	—	6	0	2	Moderate
AU-009	Yes	Yes	Yes	Unclear	Unclear	Unclear	Yes	Yes	—	—	—	—	—	5	0	3	Moderate
AU-010	Yes	Yes	Yes	Yes	Yes	Yes	Yes	Yes	—	—	—	—	—	8	0	0	Low
AU-011	Yes	Yes	Yes	Yes	Unclear	Unclear	Yes	Yes	—	—	—	—	—	6	0	2	Moderate
AU-012_STUDY1	Unclear	Unclear	Unclear	No	No	No	Yes	Unclear	Unclear	Yes	Yes	Yes	Yes	5	3	5	High
AU-012_STUDY2	Unclear	Unclear	Unclear	No	No	No	Yes	Unclear	Unclear	Yes	Yes	Yes	Yes	5	3	5	High
AU-013	Yes	Unclear	Yes	Yes	Unclear	Unclear	Yes	Yes	—	—	—	—	—	5	0	3	Moderate
AU-014	Yes	Yes	Yes	Yes	Unclear	Unclear	Yes	Yes	—	—	—	—	—	6	0	2	Moderate
AU-015	Yes	Unclear	Yes	Yes	Unclear	Unclear	Yes	Yes	—	—	—	—	—	5	0	3	Moderate
AU-016	Yes	Yes	Yes	Yes	Yes	Yes	Yes	Yes	—	—	—	—	—	8	0	0	Low
AU-017	Yes	Yes	Yes	Yes	Unclear	Unclear	Yes	Yes	—	—	—	—	—	6	0	2	Moderate
AU-018	Yes	Unclear	Yes	Yes	Unclear	Unclear	Yes	Yes	—	—	—	—	—	5	0	3	Moderate
AU-019	Yes	Yes	Yes	Yes	Unclear	Yes	Yes	Yes	—	—	—	—	—	7	0	1	Low
AU-020	Yes	Unclear	Yes	Yes	Unclear	Unclear	Yes	Yes	—	—	—	—	—	5	0	3	Moderate
AU-021	Yes	Yes	Yes	Yes	Yes	Yes	Yes	Yes	—	—	—	—	—	8	0	0	Low
AU-022	Yes	Yes	Yes	Yes	Unclear	Unclear	Yes	Yes	—	—	—	—	—	6	0	2	Moderate
AU-023	Yes	Unclear	Yes	Yes	Yes	Yes	Yes	Yes	—	—	—	—	—	7	0	1	Low
AU-024_STUDY1	Yes	Yes	No	Unclear	Unclear	Unclear	Unclear	Yes	Yes	No	Yes	—	—	5	2	4	High
AU-024_STUDY2	Yes	Yes	No	Yes	Yes	No	Unclear	Yes	No	No	Yes	—	—	6	4	1	High
AU-025	Yes	Yes	Yes	Yes	Unclear	Unclear	Unclear	Yes	—	—	—	—	—	5	0	3	Moderate
AU-026	Yes	Yes	Yes	Yes	Unclear	Unclear	Yes	Yes	—	—	—	—	—	6	0	2	Moderate
AU-027	Yes	Yes	Yes	Yes	Unclear	Yes	Yes	Yes	—	—	—	—	—	7	0	1	Low
AU-028	Yes	Yes	Yes	Yes	Unclear	Unclear	Yes	Yes	—	—	—	—	—	6	0	2	Moderate
AU-029	Yes	Yes	Yes	Yes	Yes	Yes	Yes	Yes	—	—	—	—	—	8	0	0	Low
AU-030	Yes	Yes	Unclear	Yes	Unclear	Unclear	Yes	Yes	—	—	—	—	—	5	0	3	Moderate

Appraisal unit	Q1	Q2	Q3	Q4	Q5	Q6	Q7	Q8	Q9	Q10	Q11	Q12	Q13	Yes	No	Unclear	Overall
AU_FINAL_SYSREV_001	Yes	Yes	Yes	Yes	Unclear	Unclear	Yes	Yes	—	—	—	—	—	6	0	2	Moderate
AU_FINAL_SYSREV_002	Unclear	Yes	No	Yes	Yes	No	Yes	Yes	Yes	Yes	Yes	—	—	8	2	1	Moderate
AU_FINAL_SYSREV_003	Yes	Yes	Unclear	Yes	Yes	Yes	Yes	Yes	—	—	—	—	—	7	0	1	Low
AU_FINAL_SYSREV_004	Yes	Yes	Unclear	Unclear	Yes	Yes	Unclear	Yes	—	—	—	—	—	5	0	3	Moderate
AU_FINAL_SYSREV_005	Unclear	Yes	No	Yes	Yes	No	Yes	Yes	Yes	Unclear	Yes	—	—	7	2	2	Moderate
AU_FINAL_SYSREV_006	Yes	Yes	Yes	Yes	Unclear	Unclear	Yes	Yes	—	—	—	—	—	6	0	2	Moderate
AU_FINAL_SYSREV_007	Unclear	Yes	Yes	Yes	Yes	Yes	Yes	Yes	—	—	—	—	—	7	0	1	Low
AU_FINAL_SYSREV_008	Yes	Yes	Yes	Yes	Unclear	Unclear	Yes	Yes	—	—	—	—	—	6	0	2	Moderate
AU_FINAL_SYSREV_009	Yes	Yes	Yes	Yes	Yes	Yes	Yes	Yes	—	—	—	—	—	8	0	0	Low
AU_FINAL_SYSREV_010	Unclear	Yes	Yes	Yes	Yes	Yes	Yes	Yes	—	—	—	—	—	7	0	1	Low
AU_FINAL_SYSREV_011	Yes	Yes	Yes	Yes	Unclear	Unclear	Yes	Yes	—	—	—	—	—	6	0	2	Moderate
AU_FINAL_SYSREV_012	Unclear	Unclear	Yes	Yes	Unclear	Unclear	Yes	Yes	—	—	—	—	—	4	0	4	High
AU_FINAL_SYSREV_013	Unclear	Yes	Unclear	Yes	Unclear	Unclear	Yes	No	—	—	—	—	—	3	1	4	High
AU_FINAL_SYSREV_014	Yes	Yes	Yes	Yes	Unclear	Unclear	Yes	Yes	—	—	—	—	—	6	0	2	Moderate

Supplementary Table S2c. Review-derived proportional classification thresholds

JBI checklist	Items	Low	Moderate	High
Analytical cross-sectional	8	7–8	5–6	0–4
Cohort	11	10–11	7–9	0–6
Randomized controlled trial	13	12–13	9–11	0–8

Supplementary Table S2d. Overall RoB distributions

Denominator	Low	Moderate	High	Unclear	Total
All appraisal units	12	28	6	0	46
Primary-meta appraisal units	7	14	0	0	21
Narrative/nonprimary appraisal units	5	14	6	0	25

Supplementary Table S3. GRADE certainty of evidence

All five outcome domains were rated Very low certainty. Evidence was observational and predominantly cross-sectional; the table documents domain-specific downgrading considerations.

Outcome domain	Studies / effects	Total sample size	Pooled r (95% CI)	95% prediction interval	I²	Risk of bias	Inconsistency	Indirectness	Imprecision	Publication bias	Final certainty	Explanation
Anxiety	10 / 12	7639	0.352 (0.273 to 0.426)	0.076 to 0.578	90.0%	Not serious	Serious	Not serious	Not serious	Not serious	Very low	Predominantly cross-sectional evidence; downgraded for substantial inconsistency (I²=90.0%). CI was relatively narrow and PI remained positive; Egger p=0.962 and trim-and-fill k0=0 did not indicate clear small-study effects.
Psychological well-being	11 / 12	5262	−0.292 (−0.377 to −0.202)	−0.557 to 0.027	89.7%	Serious	Serious	Serious	Serious	Not serious	Very low	Moderate RoB predominated; heterogeneity was high; the prediction interval crossed the null; measures and samples varied. Egger p=0.301 and trim-and-fill k0=0.
Depression	5 / 6	4939	0.351 (0.162 to 0.515)	−0.143 to 0.705	95.2%	Not serious	Serious	Not serious	Serious	Unable to assess	Very low	Very high heterogeneity and a prediction interval crossing the null; sparse Low-RoB evidence (k=1); publication bias not assessed because k<10.
Life satisfaction	5 / 5	2524	−0.253 (−0.367 to −0.131)	−0.491 to 0.021	79.8%	Not serious	Serious	Not serious	Serious	Unable to assess	Very low	High heterogeneity, a prediction interval crossing the null, and sparse Low-RoB evidence (k=2); publication bias not assessed because k<10.
Psychological distress	5 / 5	2037	0.506 (0.368 to 0.621)	0.166 to 0.738	86.5%	Serious	Serious	Serious	Serious	Unable to assess	Very low	Observational/cross-sectional evidence with residual confounding; high inconsistency (I²=86.5%); only five effects; four of five studies from Türkiye; one Italian sample; publication bias not assessed because k<10.

Psychological distress: $k = 5$, pooled $r = 0.506$ (95% CI 0.368–0.621), $I^2 = 86.5\%$, 95% prediction interval 0.166–0.738; four of five contributing studies were from Türkiye, publication bias could not be assessed because $k < 10$, and residual confounding remained plausible.

Supplementary Table S4. Final sample-overlap adjudication

Potential non-independence was assessed from the recorded authorship, recruitment, sample, demographic, timing, ethics, platform, and study-context information. No confirmed duplicate primary sample was identified. The table reports the final adjudication without adding new analyses.

Study/report pair	Basis for possible overlap	Final overlap judgment	Primary-analysis handling	Existing sensitivity analysis / impact	Final interpretation
Kaya et al. 2025 (N=496) / Kaya et al. 2025 (N=432)	Same first author; different age groups, coauthors, ethics information, and sample sizes.	Likely independent	Both retained in psychological well-being.	No pair-specific numerical sensitivity analysis was reported.	Samples were treated as distinct; shared authorship remains an interpretive consideration.
Kaya et al. 2025 (N=496) / Kaya et al. 2025 (N=415)	Same first author; different populations, ethics information, recruitment, and outcome domains.	Likely independent	Both retained in their respective domains.	No within-domain pair-specific numerical analysis was applicable.	Different outcome domains precluded double-counting within a primary pool.
Kaya et al. 2025 (N=432) / Kaya et al. 2025 (N=415)	Same first author and similar demographics; different ethics information, recruitment, and outcome domains.	Possible overlap, unconfirmed	Both retained in their respective domains.	No within-domain pair-specific numerical analysis was applicable.	Potential shared recruitment does not duplicate an effect within a primary domain.
Taskin et al. 2024 / Kütük et al. 2025	Shared institution and coauthor; different populations and mean ages.	Likely independent	Both retained in psychological well-being.	No pair-specific numerical sensitivity analysis was reported.	Differing recruitment characteristics supported treatment as distinct samples.
Satıcı et al. 2023 (Study III) / Taskin et al. 2024	Shared author; different institutions and recruitment frames.	Likely independent	Both retained in psychological well-being.	No pair-specific numerical sensitivity analysis was reported.	The samples were treated as distinct.
Satıcı et al. 2023 (Study III) / Kütük et al. 2025	Shared author; different institutions, first authors, sample sizes, and ages.	Likely independent	Both retained in psychological well-being.	No pair-specific numerical sensitivity analysis was reported.	The samples were treated as distinct.
McCutcheon et al. 2024 / Shabahang et al. 2024	Overlapping authors and institutions; United States subsamples had similar sizes and mean ages, whereas Iranian subsamples differed in sex distribution.	Possible overlap, unconfirmed	All eligible effects retained.	No pair-specific numerical sensitivity analysis was reported.	Results involving these author-linked samples should be interpreted cautiously.
Shabahang et al. 2023 / Shabahang et al. 2024	Same first author; different recruitment, data-collection periods, and sex distributions.	Likely independent	Both retained in anxiety.	No pair-specific numerical sensitivity analysis was reported.	Differing sample characteristics supported independence.
McCutcheon et al. 2024 / Shabahang et al. 2023	Shared authors; different recruitment and sample sizes.	Likely independent	Eligible effects retained across primary domains.	No pair-specific numerical sensitivity analysis was reported.	Differing recruitment supported treatment as distinct samples.
Kurtça et al. 2025 [N=553; GAD-7] / Türk-Kurtça & Kocatürk 2025 [N=443; trait anxiety]	Same first author under different bibliographic forms and overlapping variables; different sample sizes and anxiety constructs.	Possible overlap, unconfirmed	Both retained in the main anxiety analysis.	Main r=0.352. Excluding the N=443 trait-anxiety report: r=0.364 (95% CI 0.283–0.441; I^2=89.7%; prediction interval 0.091–0.586). Excluding the N=553 GAD-7 report: r=0.353 (95% CI 0.265–0.435; I^2=91.0%; prediction interval 0.054–0.594).	Alternate exclusions did not materially change the direction or interpretation.
Kartol et al. 2025 / Kartol et al. 2023	Same research team and post-earthquake context; different sample sizes.	Possible overlap,	The 2025 report remained narrative/nonprimary; the	Narrative-only / no primary-meta impact.	No duplicate primary effect was introduced.

Study/report pair	Basis for possible overlap	Final overlap judgment	Primary-analysis handling	Existing sensitivity analysis / impact	Final interpretation
		unconfirmed	2023 report remained in primary pools.		
Kartol et al. 2023 / Kaya et al. 2025 (N=415)	Different author teams and recruitment; similar post-earthquake context.	Likely independent	Both retained in their eligible primary domains.	No pair-specific numerical sensitivity analysis was reported.	Contextual similarity alone was not treated as sample duplication.
Ulaş et al. 2026 / Kartol et al. 2023	Different author teams, populations, and outcome domains.	Likely independent	Ulaş remained quantitative/nonprimary; Kartol remained in primary pools.	Narrative/nonprimary / no within-domain primary-meta impact.	No duplicate primary effect was introduced.
Ulaş et al. 2026 / Satıcı et al. 2023	Same institution; different populations, recruitment, and outcome domains.	Likely independent	Ulaş remained quantitative/nonprimary; Satıcı remained in primary pools.	Narrative/nonprimary / no within-domain primary-meta impact.	No duplicate primary effect was introduced.
Ulaş et al. 2026 / Canoğulları 2025	Same institution and broadly similar ages; different outcome domains.	Possible overlap, unconfirmed	Ulaş remained quantitative/nonprimary; Canoğulları remained in psychological distress.	Narrative/nonprimary / no within-domain primary-meta impact.	No duplicate primary effect was introduced.
Satıcı et al. 2023 (Study III) / Canoğulları 2025	Same institution and overlapping timeframe; different recruitment.	Possible overlap, unconfirmed	Both retained in psychological distress.	No pair-specific numerical sensitivity analysis was reported.	Institutional linkage was retained as a limitation.

Supplementary Figure S1. Study-level forest plots for the five primary outcome domains

Panels A–E show the 40 primary effects for anxiety, psychological well-being, depression, life satisfaction, and psychological distress. Squares denote Pearson correlations and diamonds denote eligible Spearman correlations. For Soraci et al. (2025), Panels A–D use the Spearman correlations reported in the main article, whereas Panel E uses the Pearson correlation reported in Supplementary Table S5. Individual 95% confidence intervals were derived from extracted Fisher-z values and standard errors. Filled orange diamonds show the random-effects pooled correlations and 95% confidence intervals; grey horizontal lines show 95% prediction intervals. All five GRADE ratings are Very low.

Panel A. Anxiety

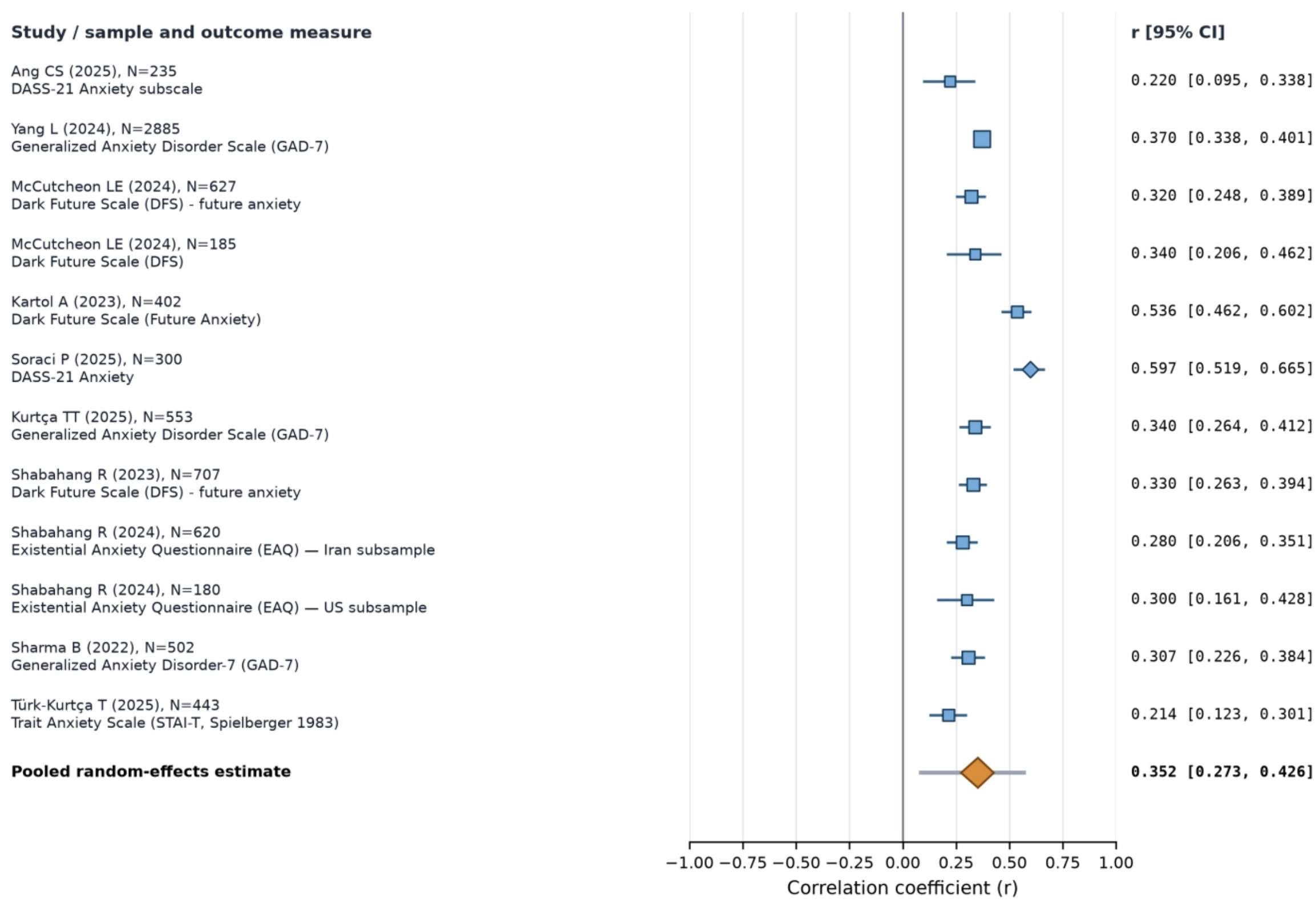

k=12 effects; I^2=90.0%; 95% prediction interval [0.076, 0.578]; GRADE certainty: Very low.

Panel B. Psychological well-being

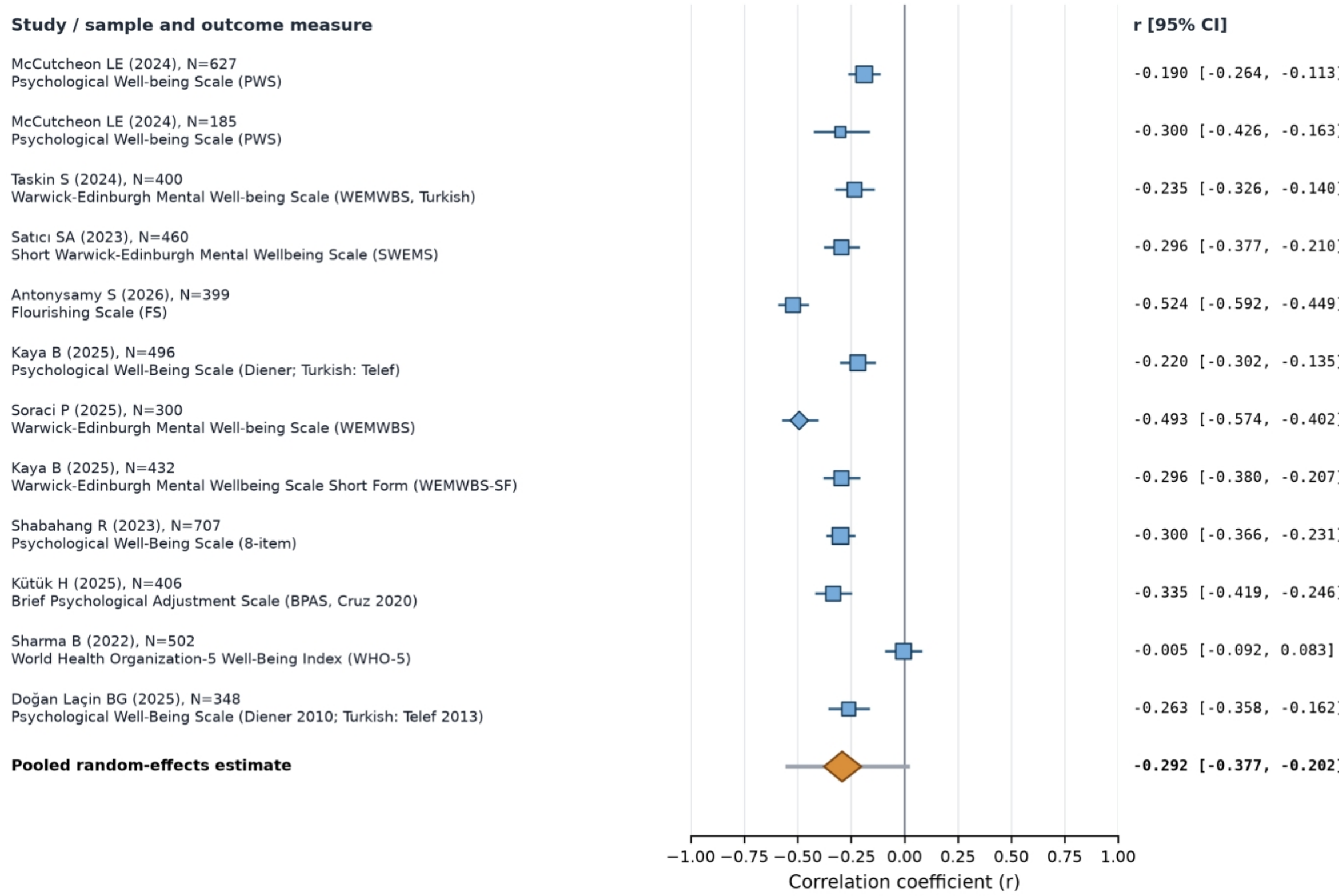

k=12 effects; I²=89.7%; 95% prediction interval [-0.557, 0.027]; GRADE certainty: Very low.

Panel C. Depression

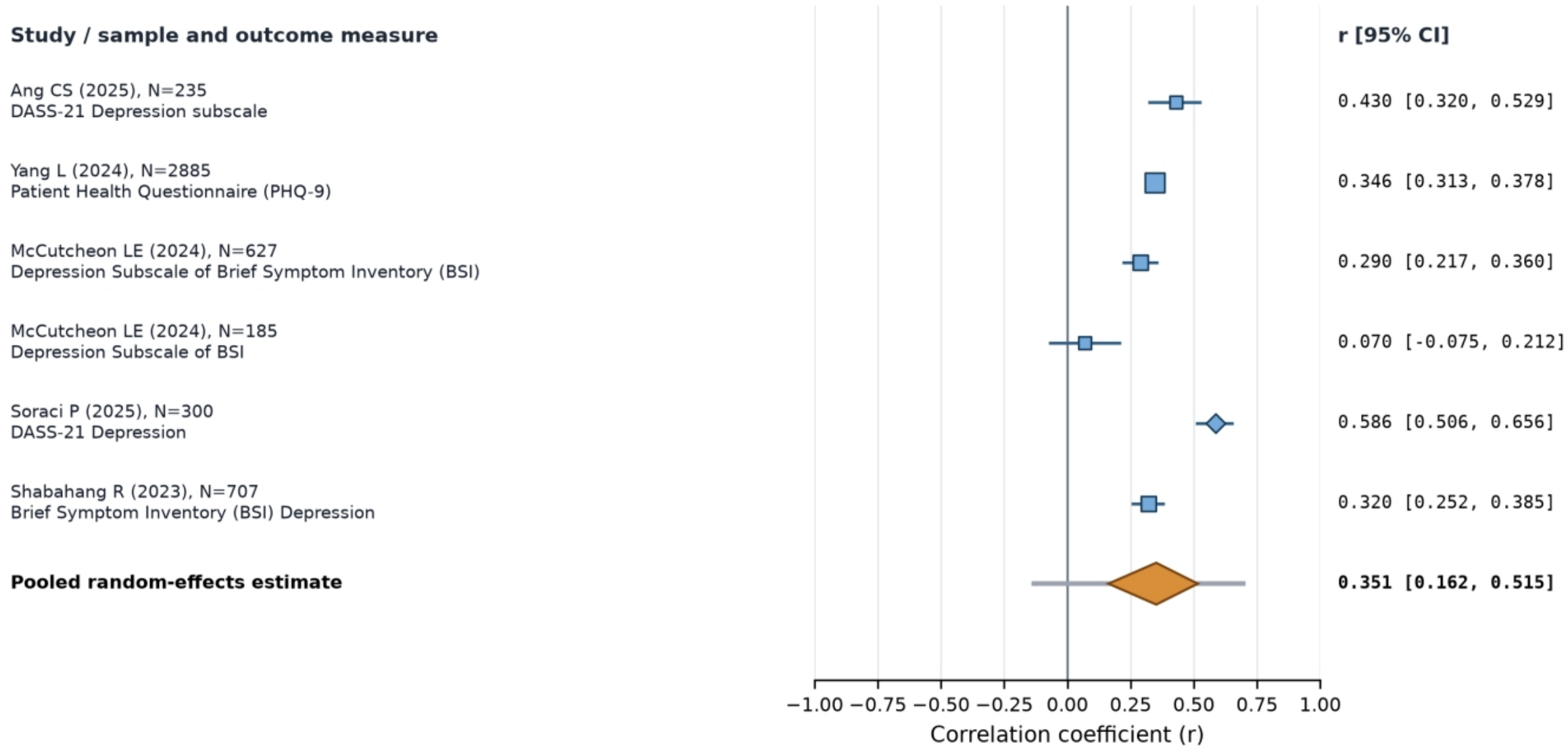

k=6 effects; I²=95.2%; 95% prediction interval [-0.143, 0.705]; GRADE certainty: Very low.

Panel D. Life satisfaction

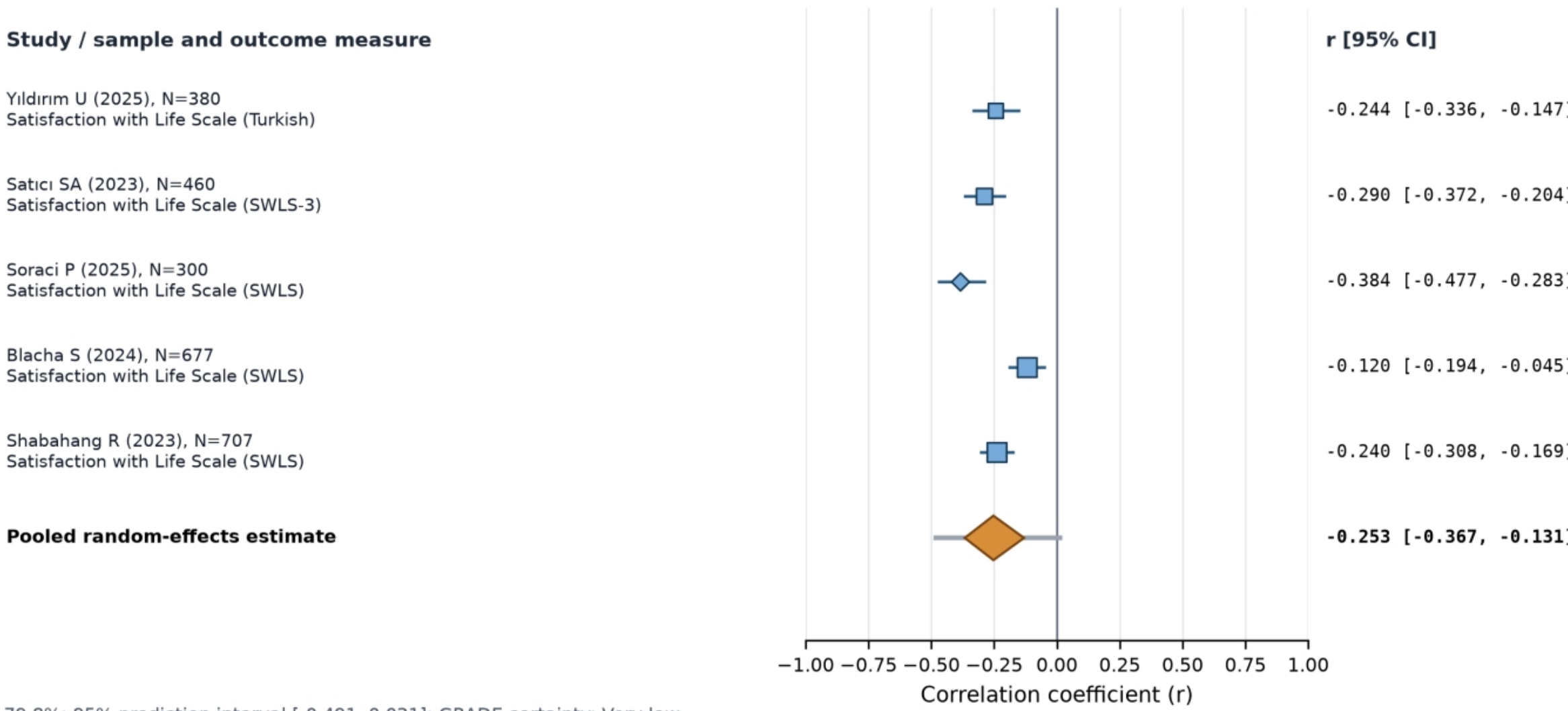

k=5 effects; I²=79.8%; 95% prediction interval [-0.491, 0.021]; GRADE certainty: Very low.

Panel E. Psychological distress

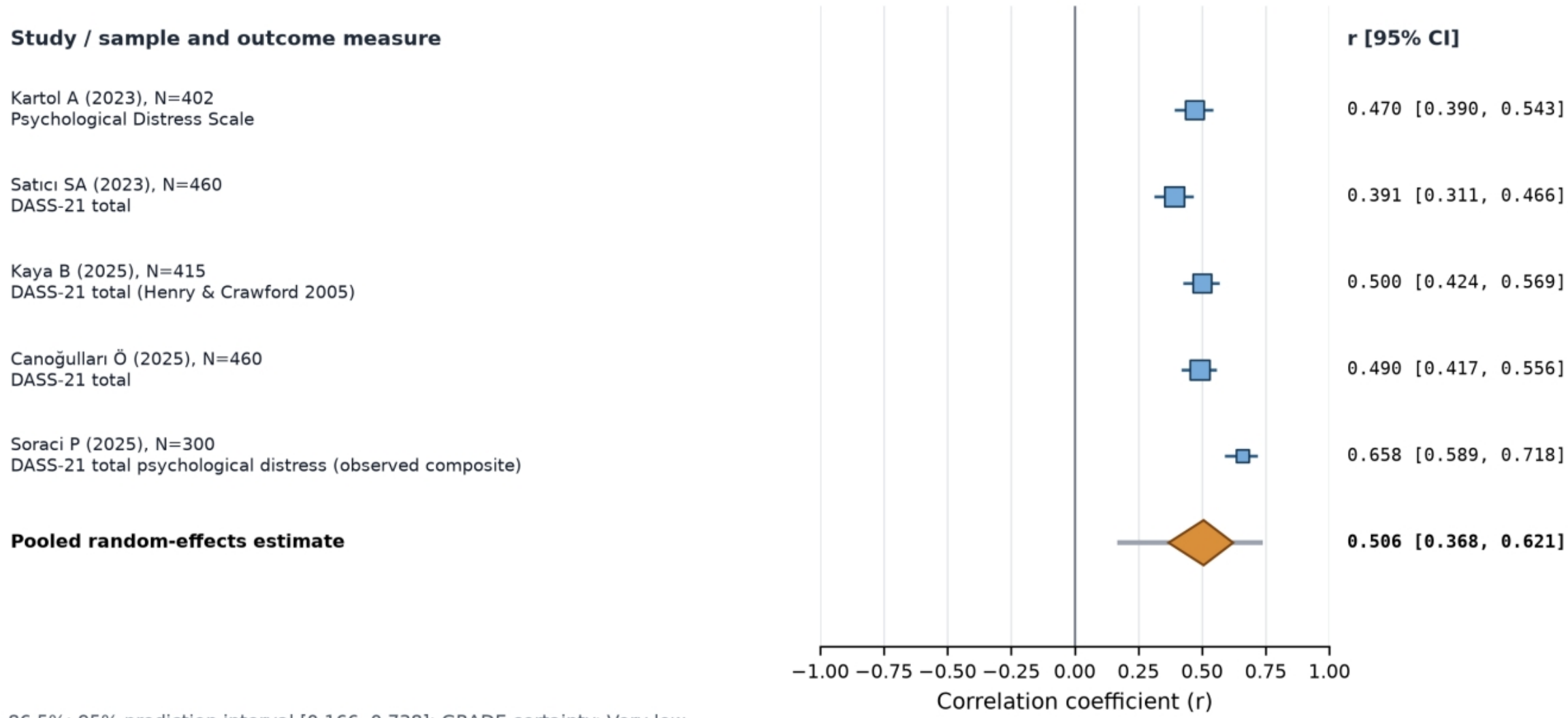

k=5 effects; I²=86.5%; 95% prediction interval [0.166, 0.738]; GRADE certainty: Very low.
